# Multifunctional microneedle-mediated photothermo-gas-ion synergic therapy accelerates MRSA infacted diabetic wound healing

**DOI:** 10.1016/j.mtbio.2025.101903

**Published:** 2025-05-24

**Authors:** Shihao Deng, Yunhao Tai, Chenxu Liu, Kenan Sun, Shaoze Lan, Liu Yang, Canming Ye, Li Huang, Runhuai Yang, Haisheng Qian, Jun Li

**Affiliations:** a Department of Orthopedics, The Second Affiliated Hospital of Anhui Medical University, Hefei, 230601, China; b Institute of Orthopedics, Research Center for Translational Medicine, The Second Affiliated Hospital of Anhui Medical University, Hefei, 230601, China; c School of Biomedical Engineering, Anhui Provincial Institute of Translational Medicine, Anhui Medical University, Hefei, 230032, China; d School of Basic Medicine, Bengbu Medical University, Bengbu, 233030, China

**Keywords:** Microneedle photothermo-gas-ion MXenes MRSA wound healing

## Abstract

Chronically high blood glucose levels in diabetic wounds form a complex microenvironment, and this microenvironment is represented by hypoxia, infection, and inflammation. Such an environment significantly impedes the wound healing cascade. Accordingly, monotherapeutic approaches have proven inadequate in addressing the complex nature of these wounds. Herein, we report the development of a reactive system comprising in situ generated glucose oxidase (GOx), Ta_4_C_3_ MXenes, and zinc sulfide nanoparticles (ZnS NPs). This system is integrated in a bilayer microneedle platform for sustained delivery. These microneedles facilitate synergistic photothermal therapy and PH responsive gas-ion therapy and also exhibit robust ROS scavenging properties. In addition, they demonstrate favorable mechanical characteristics and biocompatibility. Firstly, the inclusion of GOx effectively counteracts the negative effects of the high-sugar microenvironment. Secondly, the microneedles employ PH-responsive Zn^2+^, H_2_S release, and photothermal therapy to disrupt the structural integrity of Methicillin-Resistant Staphylococcus Aureus (MRSA) and dismantle the dense biofilm produced by this pathogen. The sustained release of MXenes offers broad-spectrum Reactive Oxygen Species (ROS) scavenging, and this process mitigates cellular apoptosis. In addition, these microneedles also enhance cell proliferation, migration, and angiogenesis *in vitro* effectively. Finally, *in vivo* studies utilizing type Ⅱ diabetic mice proved that these microneedles have photothermal antibacterial effectiveness and demonstrate their capacity to regulate inflammatory factors, promote angiogenesis and collagen deposition, and finally expedite tissue repair and regeneration, which offers a novel therapeutic strategy for the treatment of infected diabetic wounds.

## Highlights

• The three major characteristics of Ta_4_C_3_ MXenes are fully utilized (high specific surface area, excellent photothermal conversion efficiency and ROS removal performance).
• GOx lowers the pH of the microenvironment by lowering blood sugar and producing H^+^.
• ZnS nanoparticles have PH-responsive gas release and Zn^2+^ ion release and have been developed for antibacterial and anti-inflammatory purposes.
• GOx@ZT microneedles cooperate with photothermo-gas-ion therapy to treat infectious diabetic wounds.

## Graphical abstract

**Figure:**
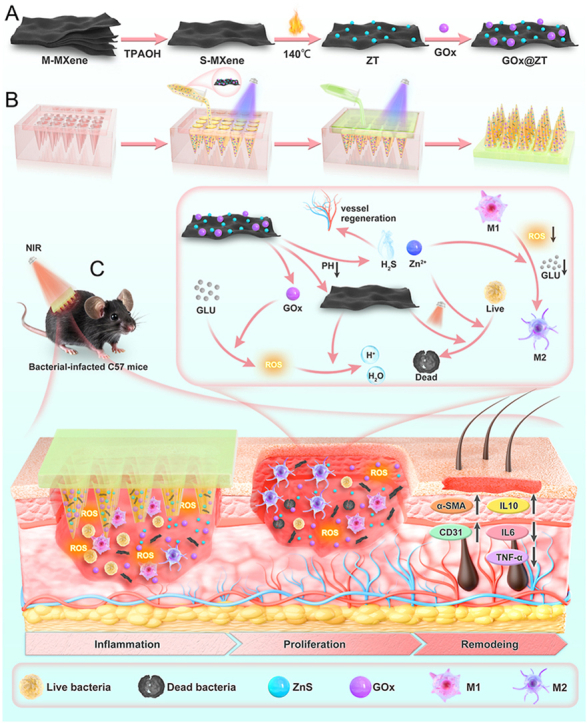

## 1 Introduction

Global statistics indicate 537 million individuals currently live with diabetes, with an estimated 19 %–34 % expected to develop diabetic foot ulcers (DFU) during their lifetime [1]. These ulcers often prove resistant to spontaneous healing. As the disease progresses, deterioration can ensue, potentially leading to amputation and inflicting significant physical and psychological burdens upon affected individuals [2,3]. The complex microenvironment characterizing diabetic wounds plays a central role in DFU formation. A high-sugar, low-oxygen environment exacerbates the production of Extracellular Polymeric Substances (EPS), thereby facilitating the development of bacterial biofilms, which significantly impede the penetration of antibacterial agents. Therefore, the effectiveness of antibiotics in treating infected DFU is frequently compromised [[4], [5], [6]]. Moreover, in the high-sugar microenvironment of infected wounds, the immune response can generate an overabundance of ROS such as superoxide anions (O^2−^), hydroxyl radicals (-OH), and hydrogen peroxide (H_2_O_2_) [7]. This oxidative stress contributes to macrophage phenotypic imbalances, perpetuating inflammatory responses, causing mitochondrial dysfunction, impairing angiogenesis, and promoting the formation of advanced glycation end products (AGEs) [[8], [9], [10]]. The absence of consistently effective treatments for DFU attributable to drug-resistant bacterial infections represents a serious and pressing clinical challenge [11]. Considering this urgency, the development of non-antibiotic therapeutic strategies for long-term and stable diabetic wound management is crucial for improving outcomes in individuals with DFU.

MXene-based materials have gained popularity in biological applications with two-dimensional nanostructure, exceptional physical and chemical properties, and biocompatibility. In addition, recent research indicates that MXene-based materials exhibit broad-spectrum reactive oxygen species ROS scavenging activity. Compared with biological enzymes, they demonstrate more robust and sustained enzyme-like activity in the complex diabetic microenvironment [12,13]. Therefore, the term "MXenzyme" has been introduced and employed to eliminate ROS to promote wound skin regeneration, fatty liver disease and myocardial infarction, among other diseases [[14], [15], [16], [17], [18], [19], [20]]. Ta_4_C_3_ nanotablets are members of the MXene family and represent a strong candidate for photoacoustic PA contrast agents due to their high extinction coefficient, excellent photothermal conversion efficiency (η = 44.7 % at 808 nm), and biocompatibility [21,22]. Numerous research groups have utilized MXenes for Photothermal Therapy (PTT) to combat infection, either through sustained release or surface modification of the material surface. Moreover, the extensive specific surface area and unique size effects of Ta_4_C_3_ nanosheets enable the construction of nanoplatforms and allow modification with specific small molecules, antibodies, or sequence structures, which yield nanomaterials with specialized properties [23]. Xiaoge Zhang et al. capitalized on this characteristic by chemically conjugating GOx and Chloroperoxidase (CPO) to Ti_2_C_3_ MXenes nanochips to conduct tumor Enzyme Dynamic Therapy (EDT), phototherapy, and oxygen-depleted activated chemotherapy [24]. Despite the numerous advantages of phototherapy, some clinical challenges exist and continue to face some challenges. The restricted tissue penetration depth of light remains a significant concern, and potential damage to healthy tissue also poses risks. Mild Photothermal Therapy (mPTT) at temperatures below 50 °C reduces adverse tissue effects and often necessitates higher temperatures for complete biofilm eradication, which can cause damage to the wound and adjacent tissue [[25], [26], [27]].

Critically, current multifunctional biomaterials typically address only a limited number of impediments to healing in recalcitrant diabetic foot ulcers DFUs [28]. This limitation hinders comprehensive treatment. To circumvent the drawbacks of photothermal therapy, the development of multimodal combination therapy offers a promising alternative. ZnS NPs is a novel class of Class Ⅱ-Ⅳ semiconductor metal chalcogenide zero-dimensional nanomaterials with favorable biocompatibility, can be fully integrated with the MXenes nanoplatform. In the acidic microenvironment of the biofilm, they release H_2_S gas and Zn^2+^ ions [29,30]. Zn^2+^, a well-established promoter of angiogenesis and antibacterial activity, modulates macrophage phenotype. This includes shifting from the pro-inflammatory M1 phenotype to the pro-healing M2 phenotype. This shift is achieved by inhibiting the NF-κB signaling pathway and activating the Janus Kinase Signal Transducer and Activator of Transcription (JAK-STAT) signaling pathway. These actions cultivate an immunoregulatory microenvironment conducive to wound repair [31,32]. In addition, Zn^2+^ upregulates vascular endothelial growth factor VEGF expression and positively affects fibroblast activation and new blood vessel formation during wound healing [33]. This upregulation promotes scar-free repair. Hydrogen sulfide (H_2_S) demonstrates promise in anti-inflammatory and antioxidant therapies [34]. Its diverse physiological effects include ion channel regulation, modulation of inflammatory responses, promotion of angiogenesis, and highly permeable disruption of bacterial biofilms. These effects make it relevant in the inflammatory microenvironment and oxidative damage characteristic of various diseases. These include intestinal inflammation, chronic renal failure, myocardial ischemia-reperfusion injury, and ischemic stroke [[35], [36], [37], [38]]. Research indicates that H_2_S not only neutralizes ROS and Reactive Nitrogen Species (RNS), but also safeguards endothelial cells against high-sugar-induced apoptosis and enhances diabetic vasculopathy [39]. H_2_S further promotes VEGF and accelerates angiogenesis. Simultaneously, H_2_S disrupts bacterial biofilms and directly kills pathogens. It reduces the level of pro-inflammatory factors by adjusting the balance of macrophage polarization (M1/M2 ratio) while increasing anti-inflammatory factors (such as IL-10, TGF-β) [34]. This process further optimizes the diabetic wound microenvironment. Nevertheless, H_2_S has a short half-life and its local concentration is difficult to regulate. Conventional exogenous donors such as sodium hydrosulfide (NaHS) and sodium sulfide (Na_2_S), while capable of rapid H_2_S release, exhibit release rates inconsistent with the requirements of the lesion. They can also induce cytotoxicity [40,41]. Therefore, the effective initiation and controlled release of H_2_S remains a central challenge.

**Fig. 1:**
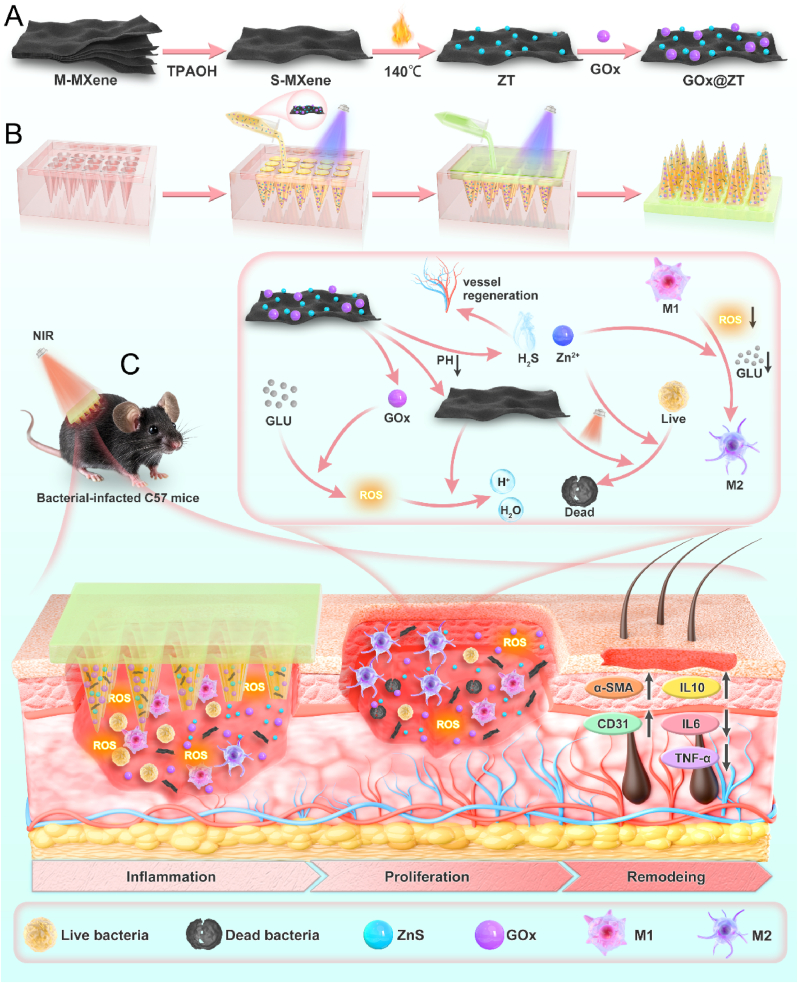
Schematic diagram of construction of GOx@ZT-MN and its application to infectious diabetic wounds. (A) GOx@ZT was generated by hydrothermal method and amidation reaction; (B) GOx@ZT-MN was synthesized by stepwise light curing; (C) GOx@ZT-MN significantly accelerates infectious diabetic wound healing by photothermo-gas-ion synergic therapy. (M-MXenes: Multilayer MXenes; S-MXenes: Single layer MXenes).

GOx can function as the trigger of the ZnS reaction system to release Zn^2+^ and H_2_S. This natural oxidoreductase exhibits high specificity in catalyzing glucose to produce gluconic acid and hydrogen peroxide and is abundant in nature [42,43]. Such a process effectively consumes surplus glucose present on wound surfaces and decreases the negative effects of the high-sugar microenvironment on wound healing. Simultaneously, the hydrogen peroxide generated through this reaction exhibits certain antibacterial capabilities and can partially restrict bacterial growth on wound surfaces [[44], [45], [46]]. Nevertheless, this reaction presents certain limitations. The generation of hydrogen peroxide worsens the buildup of ROS in diabetic wounds, inflicting further harm to wound tissues, while the accumulation of products from enzyme-catalyzed reactions may decrease the reaction rate [47,48]. Moreover, considering that the microenvironment of diabetic wounds is complex and diverse, depending exclusively on GOx effects proves insufficient for a comprehensive solution to the problem. Various complications including vascular lesions, nerve damage, and immune dysfunction resulting from high sugar levels and hypoxia cannot be adequately addressed with GOx treatment alone for effective improvement. In infectious diabetic wounds, biofilm formation enables bacteria to withstand the host's immune responses and resist the effects of antibacterial medications [49,50]. Bacteria residing in biofilms maintain a relatively inactive state with minimal metabolic activity, which restricts the penetration and effectiveness of conventional antibacterial drugs [51,52]. Microneedles (MN) have significantly enhanced this situation [53,54]. As a highly innovative drug delivery tool, the penetrative action of microneedles can breach the structural integrity of biofilms [55], facilitating rapid contact between antibiotics and bacteria, thus allowing antibiotics to fulfill their role in suppressing bacterial growth and multiplication [56,57]. Besides, microneedles offer mechanical stimulation and can promote tissue regeneration [58]. Considering the strong performance of intestinal robots, a polyethylene glycol diacrylate (PEGDA) network microneedle array was employed for the delivery of the GOx@ZT system [59]. To this end, we built and manufactured GOx@ZT microneedles (GOx@ZT-MN). GOx@ZT-MN combines the benefits of dissolvable microneedles and multi-enzyme systems. It demonstrates an excellent photothermal effect and establishes a cooperative ion and gas therapeutic model. In addition, the adaptable, intelligent response mechanism contributes enhanced biocompatibility and accurate reaction control to the overall system. A mechanistic hypothesis for the reaction system is proposed as follows (Fig. 1). The microneedles breach the biofilm barrier associated with bacterial infection, deploying the nanoreaction system. GOx reacts with the high-sugar microenvironment to produce gluconic acid and hydrogen peroxide. The acidic microenvironment of the biofilm itself facilitates the liberation of Zn^2+^ and H_2_S. This occurs as gluconic acid is produced while consuming the substrate from the GOx reaction, thereby establishing a cascade reaction. During the acute infection stage, under Near-Infrared (NIR) light irradiation, Ta_4_C_3_ MXenes generates high temperatures and promotes increased H_2_S and Zn^2+^ production. Photothermal therapy also enhances bacterial membrane permeability. When it is combined with the synergistic action of Zn^2+^ and H_2_S, the treatment becomes more effective at offering PTT multimodal antibacterial treatment. In the proliferative phase, MXenzym (Ta_4_C_3_) scavenges excess ROS in the microenvironment and acts in concert with other components to remodel the immune microenvironment, thus supporting tissue growth. In *vitro* and *in vivo* studies were performed to confirm the biocompatibility and effectiveness of this design, offering a robust translational medicine approach for the multimodal treatment of drug-resistant bacterial infections in diabetic wounds.

## 2 Results and discussion

### 2.1 Synthesis and characterization of GOx@ZT

**Fig. 2:**
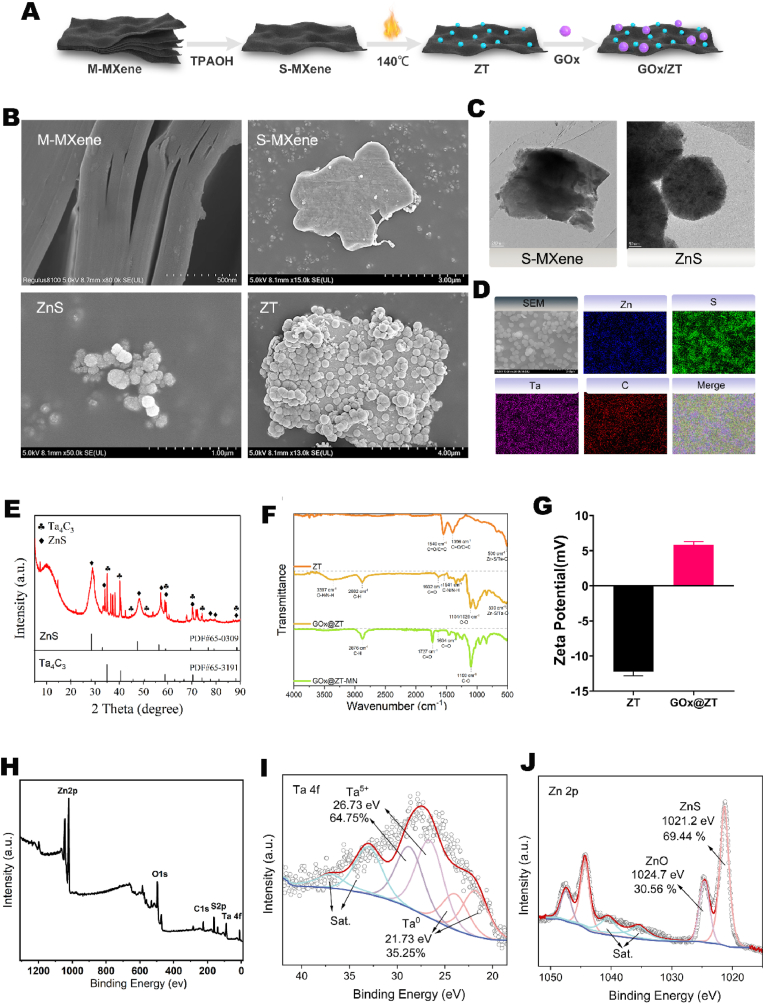
Physiochemical characterizations of GOx@ZT. (A) Pattern diagram and (B) SEM image of GOx@ZT synthesis. (M − MXenes scale bar = 500 nm, S-MXenes scale bar = 3 μm, ZnS scale bar = 1 μm, ZT scale bar = 4 μm) (C) TEM image of S-MXenes and ZnS nanoparticles. (S-MXenes scale bar = 200 nm, ZnS scale bar = 500 nm) (D) SEM-EDS image of GOx@ZT (Scale bar = 2 μm). (E) XRD of Ta_4_C_3_ MXenes and ZnS nanoparticles. (F) FTIR spectra of ZT, GOx@ZT and GOx@ZT-MN. (G) Zeta potential of ZT and GOx@ZT. (H) Full XPS survey spectra of GOx@ZT. (I) High-resolution Ta 4f XPS spectra of GOx@ZT. (J) High-resolution Zn 2p XPS spectra of GOx@ZT.

GOx@ZT was prepared through a multistep process. Firstly, a monolayer of Ta_4_C_3_ MXenes was produced and exhibited high photothermal conversion efficiency and extremely high biocatalytic activity (Fig. 2A) [60]. This began with the selective etching of aluminum from multilayer Ta_4_AlC_3_ MAX utilizing a 50 % HF solution and a tile-like stacked multilayer Ta_4_C_3_ MXenes was produced. Then, this multilayer Ta_4_C_3_ MXenes was intercalated with tetrapropylammonium hydroxide (TPAOH) to produce a sheet-like monolayer Ta_4_C_3_ MXenes (Fig. 2B and C). The ZnS/Ta_4_C_3_ (ZT) nanoplatform was then formed in situ through a hydrothermal reaction. This involved heating the monolayer Ta_4_C_3_ nanosheets with [Zn (CH_3_COO) _2_] ·2H_2_O and thiourea in an autoclave at 140 °C for 12 h, and then the mixture was processed further. The derived ZT nanoplatform was collected after washing, centrifugation, and drying. Elemental analysis through energy dispersive spectroscopy (EDS) mapping confirmed the presence of Zn, S, Ta, and C in the synthesized ZT nanoplatform (Fig.

**2B, 2D**). This composition was further corroborated by XRD analysis. Diffraction peaks (marked in blue) appeared at 28.61°, 33.24°, 47.52°, 56.74°, 69.63°, and 76.92°, and these peaks were indexed to the (111), (220), (311), (400), and (331) planes of cubic ZnS (zinc blende), respectively, and were consistent with the diffraction data of the standard card (ICCD #65–0309). Additional diffraction peaks at 35.20°, 40.75°, 59.00°, and 70.54° (marked in black) were assigned to the (111), (200), (220), and (311) planes of Ta_4_C_3_ MXenes, respectively. The preceding results confirm the successful synthesis of the ZT nanoplatform (Fig. 2E). In addition, we measured the full XPS spectrum of GOx@ZT-MN(Fig. 2H), and detected the peaks of Ta 4f (Fig. 2I), Zn 2p (Fig. 2J), S2p(Fig. S2A), and C1s(Fig. S2B) in the XPS spectrum of ZT, and the characteristic peaks of the ZT nano-platform were obtained by peak fitting. All these results indicate that the interface between ZnS and Ta_4_C_3_ was successfully combined.

Then, GOx was conjugated to the ZT nanoplatform through an amidation reaction [61,62]. The infrared spectral characteristics of the resulting GOx@ZT conjugate are presented in the Fig. 2F. Ta_4_C_3_ typically exhibits no molecular vibrational absorption peaks in the infrared spectrum, whereas the characteristic peak of ZnS arises primarily from the stretching vibration of the Zn-S bond. Therefore, the broad peak observed near 500 cm^−1^ is primarily assigned to the Zn-S stretching vibrational mode. A combined absorption peak, attributed to the O-H and N-H stretching vibrations in the GOx amide A band, appears at 3397 cm^−1^. The absorption peak at 2882 cm^−1^ corresponds to the stretching vibrations of methyl, methylene, and methine groups in the side chains of GOx amino acids. In addition, the absorption peak at 1632 cm^−1^ (amide I band) is ascribed to the C=O stretching vibration in the GOx amide group (-NH-CO-). The subtle peak at 1541 cm^−1^ is associated with the N-H bending and C-N stretching vibrations of primary and secondary amine groups in GOx (-NH-CO-). The complex absorption peak near 1459 cm^−1^ is primarily associated with the C-H stretching vibration of the methylene group, the symmetrical stretching vibration of C=O, and the C-N stretching vibration of the aromatic amino group. The absorption peak at 1020 cm^−1^ is mainly due to the C-O stretching vibration. Analysis of the XRD pattern of GOx@ZT-MN indicates characteristic peaks for both ZnS and Ta_4_C_3_, as well as a characteristic peak for GOx at 8.63° (Fig. S1). Upon the addition of GOx, the Zeta potential changes from negative to positive and indicates the GOx@ZT platform is succesfully prepared (Fig. 2G).

### 2.2 Synthesis and characterization of GOx@ZT-MN

**Fig. 3:**
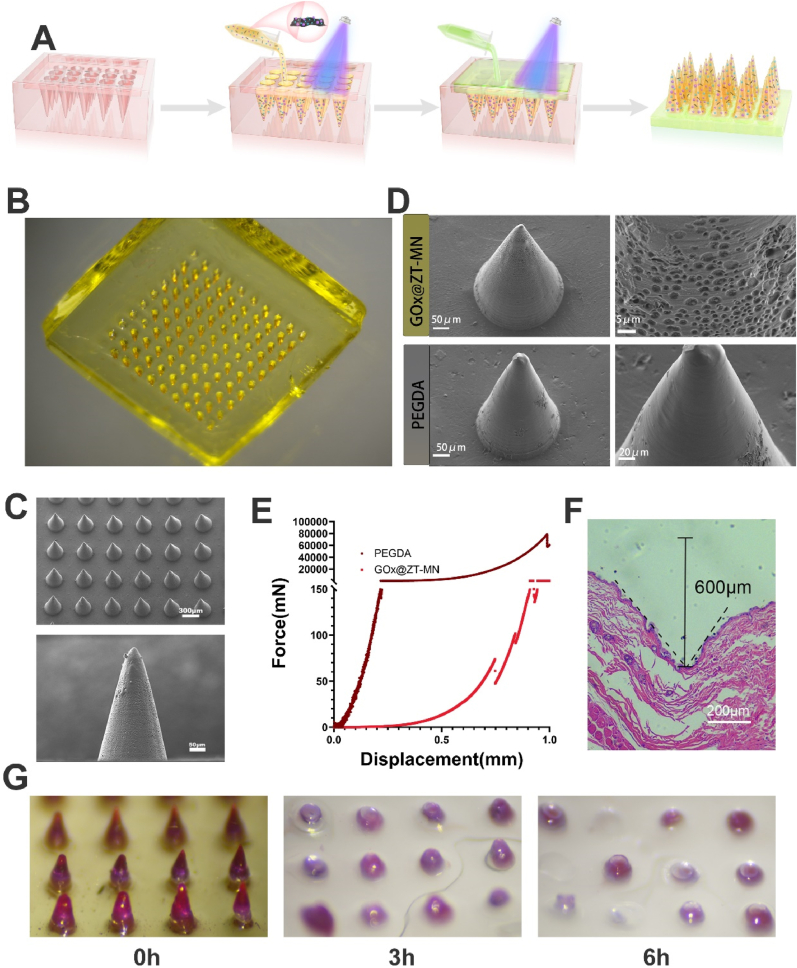
Physiochemical characterizations of GOx@ZT-MN. (A) Pattern diagram of GOx@ZT-MN synthesis. (B) Photos and (C) SEM image of GOx@ZT-MN. (Scale bar = 300 μm, 50 μm) (D) SEM image of PEGDA and GOx@ZT-MN. (GOx@ZT-MN scale bar = 50 μm, 5 μm; PEGDA scale bar = 50 μm, 20 μm) (E) Force-displacement curve of PEGDA and GOx@ZT-MN. (F) Skin penetrating test in mice. (Scale bar = 200 μm) (G) Photos of the dissolution of GOx@ZT-MN.

The network formed by rapid crosslinking of PEGDA exhibits a small, dense mesh size that presents challenges for drug delivery. To address this, polyethylene glycol PEG was incorporated as a diluent to impede PEGDA crosslinking. PEGDA, a hydrophilic polymer with favorable biocompatibility and robust mechanical properties, rapidly forms a dense, low-molecular-weight, three-dimensional network structure with small pores through photo-crosslinking. This process creates a matrix suitable for drug release. However, its poly (ether)backbone confers good hydrolytic stability [63,64]. The addition of PEG further decreases the crosslinking density of PEGDA, thus accelerating the degradation rate and

facilitating more rapid drug release [65]. The microneedle patch was manufactured utilizing a two-step photo-curing process (Fig. 3A). The microneedle tips consist of PEGDA, PEG solutions, and GOx@ZT particles, while the backing layer was formed from a mixture of adhesive acrylamide and PEGDA (Fig. 3B). Scanning electron microscopy SEM indicated that microneedles composed of the PEG/PEGDA mixture have a larger mesh structure and increased porosity (Fig. 3D). The fabricated patch incorporates a 10 × 10 microneedle array (Fig. S2). SEM imaging demonstrated that each microneedle demonstrated a conical geometry with a height of 600 μm and a base diameter of 300 μm (Fig. 3C). The mechanical strength of the GOx@ZT-MN was assessed utilizing a force-displacement curve, indicating a strength of approximately 0.72 N per needle, exceeding the minimum force necessary for skin penetration (Fig. 3E) [66]. To further test the penetration capacity of the GOx@ZT-MN, puncture studies and hematoxylin and eosin HE staining were performed on mouse skin. The results confirmed effective penetration by the GOx@ZT-MN (Fig. 3F). To better visualize microneedle dissolution, dye was incorporated into the needle tips. As depicted in the figure, after 3 h of soaking, the microneedles dissolved nearly half, with near-complete dissolution observed after 6 h. Different degrees of fading were apparent in the backing layer and needle tips. This observation suggests greater fluidity in the microneedle patch (Fig. 3G). Therefore, it is necessary to test the release profiles of individual microneedle components. Effective wound treatment requires a certain degree of adhesion from the microneedle patch.

### 2.3 Photothermal performance of GOx@ZT-MN

**Fig. 4:**
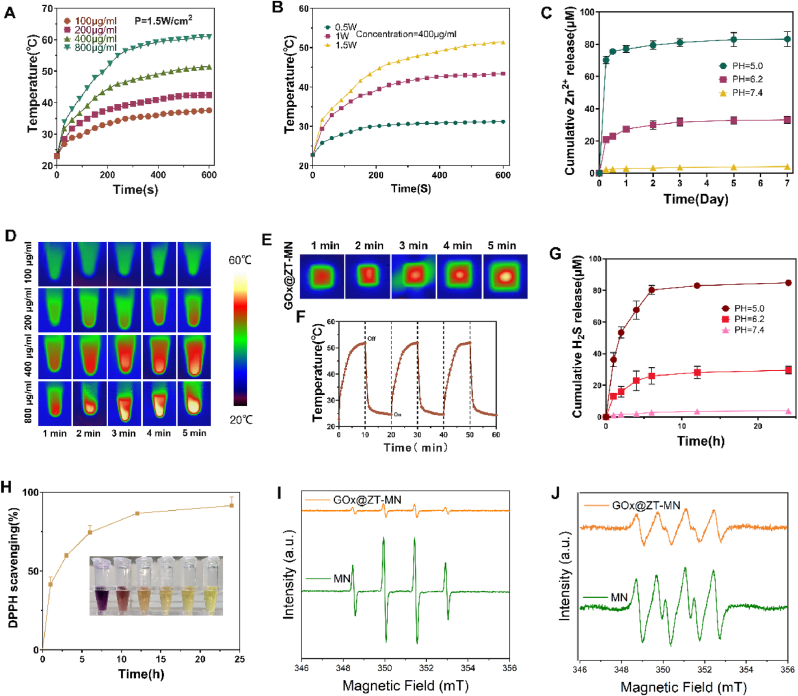
Performances of GOx@ZT-MN. (A) Temperature elevation curves and (D) thermal image of aqueous PBS solutions with various concentrations of GOx@ZT under NIR laser (808 nm, 1.5Wcm^−2^) for 600s. (B) Temperature elevation curves of various density of powerith of 400 μgml^−1^ GOx@ZT under NIR laser (808 nm) for 600s. (C) Zn^2+^ release performance of GOx@ZT-MN with different pH over a 7-day period. (E) Thermal image of 400 μgml^−1^ GOx@ZT and GOx@ZT-MN under NIR laser (808 nm, 1.5Wcm^−2^) for 5min. (F) Photothermal conversion ability of GOx@ZT-MN during 3 times of on/off NIR irradiation cycles. (G) H_2_S release performance of GOx@ZT-MN with different pH over a 24-h period. (H) Scavenging DPPH activity of GOx@ZT-MN with different pH over a 24-h period. (I) Scavenging effects of GOx@ZT-MN on ·OH and (J) O_2_^−·^ evaluated by EPR. Data are presented as mean ± SD (n = 3).

The photothermal properties of GOx@ZT were assessed as a function of concentration and laser power density to determine an appropriate concentration for microneedle fabrication. PBS solutions were irradiated with an 808 nm laser at 1.5 Wcm^−2^ for 10 min, and these solutions contained varying concentrations of GOx@ZT (100, 200, 400, 800 μgml^−1^). Fig. 4A and C demonstrate a positive correlation between GOx@ZT concentration and photothermal conversion efficiency in the PBS solutions. A temperature of 51.4 °C was achieved in the PBS solution containing 400 μgmL^−1^ GOx@ZT, and the temperature rose to 60.9 °C with 800 μgmL^−1^. To explore the effect of laser power density on photothermal conversion, the relationship between laser power density and photothermal conversion efficiency of GOx@ZT was analyzed Fig. 4B. Temperatures of 43.4 °C and 31.2 °C were observed at laser power

densities of 1 Wcm^−2^ and 0.5 Wcm^−2^, respectively. These findings indicate that temperature increases can be modulated by controlling GOx@ZT concentration and laser power density. The potential for PTT-induced damage to healthy tissues must be considered, especially above 50 °C [67]., and while acknowledging that mPTT may not achieve the effectiveness of PTT at higher temperatures, mPTT minimizes tissue damage and may even considerably enhance angiogenesis and tissue regeneration. Therefore, to optimize both antibacterial effectiveness and biosafety [68], a GOx@ZT concentration of 400 μgmL^−1^ was selected for microneedle fabrication. This concentration, in conjunction with gas therapy and the action of metal ions, enables a multi-modal treatment approach.

We evaluated the photo-thermal conversion efficiency of GOx@ZT-MN at a loading of 400 μgmL^−1^. Fig. 4D demonstrates the significant temperature increase in GOx@ZT-MN under 808 nm NIR irradiation, reaching 51.9 °C in 10 min under humid conditions. To determine the stability and reliability of this photo-thermal response, we carried out three consecutive "on-off" experiments with GOx@ZT-MN. As presented in Fig. 4E and F, GOx@ZT-MN reached ≈52 °C in 10 min and then quickly dropped back to room temperature upon cessation of irradiation. Re-exposure to NIR produced similar temperature increases, and there was no significant difference in the maximum temperatures among the three times. This behavior demonstrates the great potential of GOx@ZT-MN for long-term phototherapy applications.

### 2.4 PH-responsive release of Zn^2+^ and H_2_S from GOx@ZT-MN

ZnS nanoparticles generally have strong stability in neutral pH environments; however, they will quickly decompose into H_2_S and Zn^2+^ under acidic conditions. The low pH of the diabetic microenvironment can just allow ZnS nanoparticles to decompose into H_2_S and Zn^2+^ for wound treatment and regulation [69]. The H_2_S release behavior of the GOx@ZT-MN system, as presented in the Fig. 4G, demonstrates significant pH response characteristics. Under physiological conditions (pH = 7.4), the cumulative release rate of H_2_S in 24 h is less than 5 μM and indicates that its structure has sufficient stability and sustained-release properties. In the acidic environment (pH = 5.0), which simulates an infectious diabetic wound, H_2_S was released rapidly and reached 36.2 μM in 1 h; the cumulative release in 24 h reached an astonishing 84.9 μM. At pH = 6.2, the release rate of H_2_S is in between, the cumulative release in 24 h reached an astonishing 29.6 %, thus confirming that GOx@ZT-MN has the ability to respond accurately to the acidic microenvironment while it triggers release. We also tested the release of H_2_S *in vivo* (Fig. S5): after completing the wound modeling, GOx@ZT-MN and the blank group were used for treatment, and tissue homogenate was prepared 6 h, 12 h and 24 h. The results showed that H_2_S in the control group showed a slowly increasing trend. This difference may be related to the increase in endogenous H_2_S in mice due to stress response after modeling. However, the H_2_S level in the GOx@ZT-MN group had significantly increased (472.75 ± 14.77 μmol/g) to its peak at 6 h and remained higher than that in the control group. Similarly, the release of Zn^2+^, another product of the decomposition of ZnS nanoparticles, was evaluated. Under physiological environment (pH = 7.4), there was no obvious decomposition trend, and the release rate of Zn^2+^ was only 4.13 ± 0.14 μM in 7days. And in acidic environments (PH = 5.0), ZnS quickly decomposes, and the cumulative release exceeds 70.0 ± 1.85 μM in 6 h (Fig. 4C), then the release gradually slowed down. This microenvironment responds to Zn^2+^ and H_2_S release patterns and greatly improves the safety and effectiveness of microneedles.

### 2.5 Enzyme activity of GOx@ZT-MN

Excessive levels of ROS leads to a continuous inflammation response, necessitating effective ROS clearance from the wound site. The 1-diphenyl-2-picryl-hydrazyl (DPPH) assay, a standard measure of free radical scavenging capacity, was employed to assess the ROS scavenging potential of the nanomaterials. We predict that incorporating Ta_4_C_3_ MXenes would give microneedles the ability to clear ROS. After we co-cultured GOx@ZT-MN with the DPPH reagent, a gradual lightning of the reagent color was observed. This observation indicated a steadily increasing DPPH clearance rate that finally reached 94.35 % at 24 h. Electron Paramagnetic Resonance (EPR) spectra show that GOx@ZT-MN can effectively scavenge -OH and O_2_^−^. These results confirm the sustained and efficient ROS scavenging activity of GOx@ZT-MN (Fig. 4I and J).

In addition, the enzymatic activity of GOx in GOx@ZT-MN and GOx@ZT was evaluated at room temperature and 50 °C to meet the requirements for photothermal therapy. The results indicated that at room temperature, GOx@ZT exhibited the highest enzyme activity, with glucose levels decreasing to 51.7 % after 6 h of reaction. Initially, the GOx group demonstrated higher enzyme activity compared to the GOx@ZT-MN group. However, by the 4-h mark, the GOx@ZT-MN group surpassed the GOx group, likely due to the removal of the reaction product H_2_O_2_ by Ta_4_C_3_ MXenes. Furthermore, it was observed that the enzyme activity of the GOx@ZT-MN group was lower than that of the GOx@ZT group, which may be attributed to the sustained-release effect of the microneedles. When the reaction was conducted at 50 °C, a reduction in enzyme activity was noted in the GOx group, with 85 % of glucose remaining after 6 h. In contrast, both the GOx@ZT and GOx@ZT-MN groups consumed more glucose, leaving 61.7 % and 72.2 % residual glucose respectively. We increased the glucose concentration in the medium, and then co-cultured GOx@ZT-MN infiltration solution with L929 cells. The experimental results showed that the GOx@ZT-MN group significantly reduced the glucose concentration in the medium; in contrast, although the glucose concentration in the control group also decreased, the decrease was significantly lower than that in the experimental group. In order to further explore the impact of this phenomenon on cell proliferation, we compared cell proliferation in two different environments. The results showed that GOx@ZT-MN could significantly promote the growth of L929 cells, while the proliferation rate of cells in the control group was slower. In addition, in order to verify the effectiveness of this effect in the vivo environment, we tested the glucose concentration of wound exudates in diabetic mice. The results showed that the GOx@ZT-MN group showed significant local blood sugar lowering effect, while no other group achieved effective reduction in glucose concentration. This suggests that the association with the ZT nanoplatform effectively protected the bioactivity of GOx (Fig. S6 and S7).

### 2.6 Biocompatibility of GOx@ZT-MN

For biomaterial applications, a thorough understanding of the potential biotoxicity risks associated with Zn ^2^ and gas therapies is clarified urgently. This study systematically evaluated the biological safety of GOx@ZT-MN utilizing an *in vitro* model simulating the high-sugar microenvironment of diabetic wounds. Material toxicity was quantitatively analyzed through the CCK-8 assay in a co-culture system comprising high-sugar DMEM, L929 fibroblasts, and human umbilical vein endothelial cells (HUVECs) endothelial cells. Results demonstrated significantly higher cell survival rates in the GOx@ZT-MN, ZT-MN, Ta_4_C_3_-MN, and microneedle treatment groups compared to the negative control group (100 %), confirming that the material components still maintained good cellular compatibility in the pathological microenvironment (Fig. S8). We aimed to comprehensively verify its clinical applicability, so we carried out further evaluations of hemocompatibility and *in vivo* toxicity. Hemolysis rates for all experimental groups were lower than the clinical safety threshold (1 %), which met the blood contact requirements for medical materials (Fig. S9). We analyzed major organs (heart, liver, spleen, lung, and kidney) in mice by HE staining and identified that no abnormal phenomena such as inflammatory infiltration, tissue necrosis, or fibrosis occurred in the treatment group (Fig. S10). as well as blood routine (Fig. S11) and biochemical (Fig. S12) analysis, This indicates that the microneedles did not cause systemic toxicity during metabolism *in vivo*, which is a positive finding. Based on the above results, GOx@ZT-MN microneedles have demonstrated excellent biocompatibility in cell-blood-organ dimensions, offering key safety support for their clinical transformation.

### 2.7 In vitro antibacterial and anti-biofilm activities of GOx@ZT-MN

**Fig. 5:**
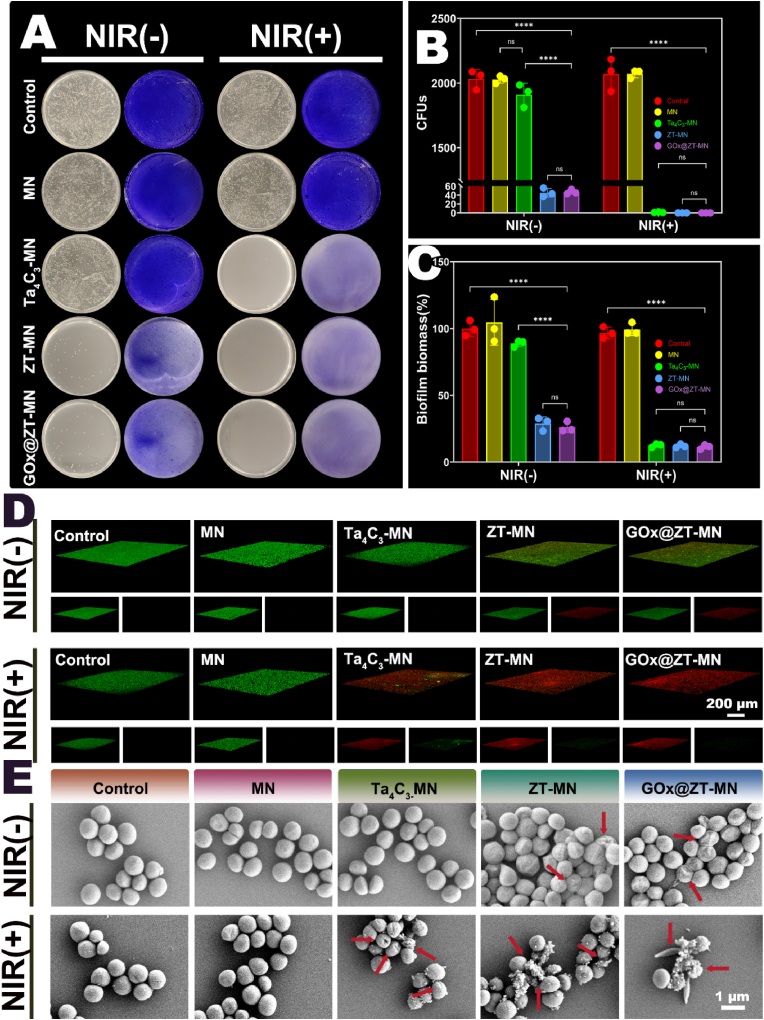
The antibacterial effects properties of different microneedles *in vitro*. (A) Macroscopic view of plates of solid medium inoculated with MRSA after immersion with microneedles, and crystal violet staining of MRSA biofilms treated with different microneedles. (B) Colony Forming Units and (C) Biofilm Biomass of MRSA treated with different microneedles. (D) DMAO/PI staining of MRSA biofilms after immersion with microneedles. (Scale bar = 200 μm) (E) SEM image of MRSA after immersion with microneedles. (Scale bar = 1 μm) NIR(+): NIR irradiation, NIR(−): No NIR irradiation. Data are presented as mean ± SD (n = 3). ∗*P* < 0.05, ∗∗*P* < 0.01, ∗∗∗*P* < 0.001, ∗∗∗∗*P* < 0.0001. ns: no significant difference. (For interpretation of the references to color in this figure legend, the reader is referred to the Web version of this article.)

The researchers considered the unique photothermal response and pH response characteristics of GOx@ZT-MN materials, hence they selected methicillin-resistant *Staphylococcus aureus* (MRSA), a prevalent multi-drug-resistant bacterium that exhibits rapid proliferative activity, as the target strain for this study. To evaluate the *in vitro* antibacterial

properties of GOx@ZT-MN, a standard plate counting method was employed, as presented in Fig. 5A and B, under both natural light and NIR irradiation respectively. Under natural light irradiation, the number of MRSA colonies treated in GOx@ZT-MN group (CFU = 47 ± 4.32) and ZT-MN group (CFU = 45.33 ± 7.93) was significantly reduced compared with other groups. The number of colonies in other groups remained at around 2000, and the antibacterial effect was not obvious. Under near infrared light, GOx@ZT-MN group (CFU≈0), ZT-MN group (CFU≈0) and Ta_4_C_3_-MN group (CFU = 1.33 ± 0.47) all demonstrated good bactericidal potential. Compared with the Ta_4_C_3_-MN group, the induction effect of GOx@ZT-MN group and ZT-MN group on bacterial death was more prominent and strongly verified the synergistic antibacterial effect between gas therapy and Zn^2+^ and phototherapy (Fig. 5A and B). Biofilm biomass was evaluated utilizing crystal violet staining. As illustrated in Fig. 5A and C, the GOx@ZT and ZT-MN groups, in the absence of near-infrared irradiation, exhibited a light purple hue and minimal biofilm coverage. Upon exposure to near-infrared irradiation, biofilm coverage was insignificant in the GOx@ZT-MN, ZT-MN, and Ta_4_C_3_-MN groups, a finding consistent with the plate count data. To further explain the extent of biofilm damage and biofilm density, samples were stained with DMAO/PI and analyzed through Confocal Laser Scanning Microscope (CLSM). Fig. 5D demonstrates dense biofilm structures in both the control and MN groups. Following near-infrared irradiation and treatment with GOx@ZT/GOx and ZT-MN, significant disruption of biofilm integrity was observed. This disruption was represented by abundant red fluorescence (representing dead bacteria), and the near-complete disappearance of green fluorescence (representing living bacteria) was also noted. Of particular note, the Ta_4_C_3_-MN group retained a small degree of biofilm coverage, while the GOx@ZT-MN and ZT-MN groups were almost entirely covered by red fluorescence. This phenomenon is attributable to the pH-responsive behavior of ZnS, which promotes the rapid release of H_2_S and Zn^2+^, thereby producing a more comprehensive osmotic sterilization effect. To gain a more detailed visualization of the effects of GOx@ZT-MN on MRSA morphology and bacterial membrane integrity, scanning electron microscope (SEM) was employed to assess the antibacterial effectiveness of the microneedles. As presented in Fig. 5E, under natural light conditions, both the control and MN groups exhibited characteristic spherical morphologies. In contrast, the GOx@ZT-MN and ZT-MN groups presented with increased surface folding and cellular shrinkage. After near-infrared irradiation, the GOx@ZT-MN, ZT-MN, and Ta_4_C_3_-MN groups displayed significant morphological deformations. These included cracking and exudate leakage, which resulted in the rapid expulsion of bacterial matrix components. These observations are in strong agreement with the preceding experimental results, further confirming the conclusion that the synergistic antibacterial strategy employing photothermal therapy, ion therapy, and gas therapy effectively compromises biofilm integrity and bacterial membrane permeability.

### 2.8 GOx@ZT-MN prevents apoptosis by scavenging ROS

**Fig. 6:**
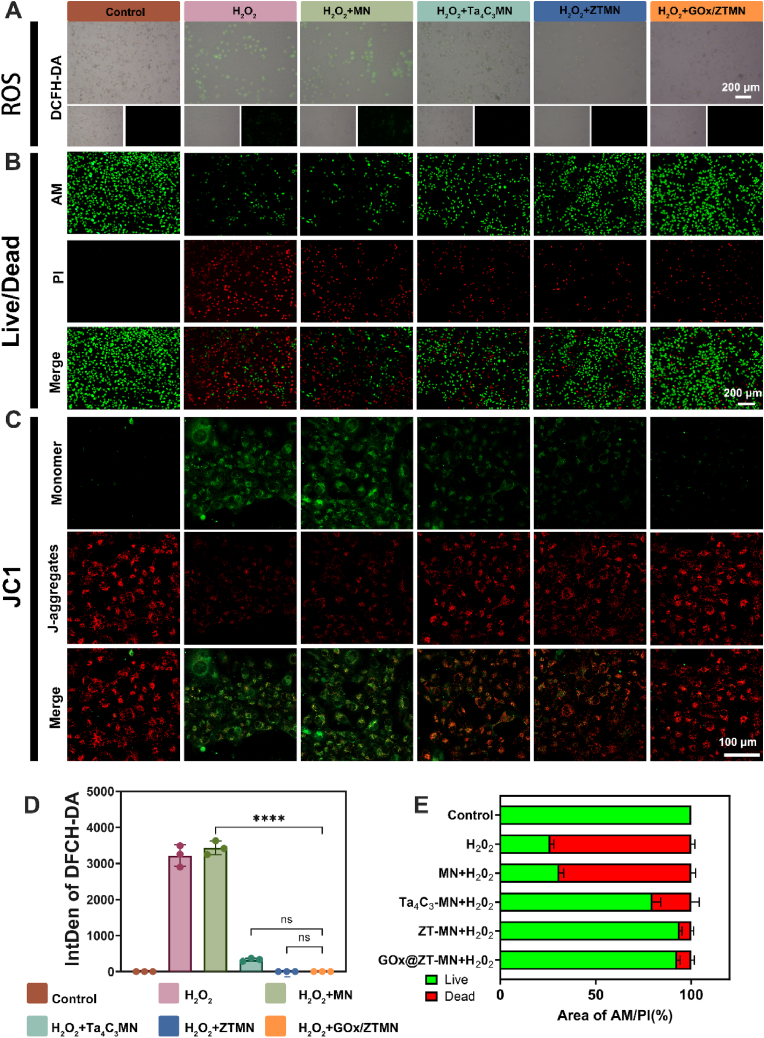
GOx@ZT-MN prevents apoptosis by scavenging ROS *in vitro*. (A) Fluorescence images and (D) quantitative graphs of intracellular ROS analysis with DCFH-DA in ROS-stimulated L929 cells after co-culture with different microneedles. (Scale bar = 200 μm) (B) Fluorescence images and (E) quantitative graphs of Live/Dead staining for ROS-stimulated L929 cells co-cultured with different microneedles. (Scale bar = 200 μm) (C)Fluorescence images of Mitochondrial membrane potential staining for ROS-stimulated L929 cells co-cultured with different microneedles. (Scale bar = 100 μm) Data are presented as mean ± SD (n = 3). ∗*P* < 0.05, ∗∗*P* < 0.01, ∗∗∗*P* < 0.001, ∗∗∗∗*P* < 0.0001. ns: no significant difference.

Excessive levels of ROS are present in diabetic wounds and contribute to immune dysfunction. To further explore the regulatory effects of GOx@ZT-MN on ROS in *in vitro* cell experiments, hydrogen peroxide (H_2_O_2_), a common ROS, was utilized as a representative. The DCFH-DA probe was employed to quantitatively evaluate ROS levels in L929 cells *in vitro*. As reported in Fig. 6A and D, the fluorescence intensity of the GOx@ZT-MN treated group was significantly reduced compared to the control and MN groups (the comprehensive optical density intensity exceeded 3000). However, no significant difference was observed between the GOx@ZT-MN, ZT-MN, and Ta_4_C_3_-MN groups. These results strongly support our previous material characterization and analysis and further confirm the excellent ROS scavenging capacity of GOx@ZT-MN. Besides, they suggest potential applications for MXene-based enzymes in the biomedical field. To explore the protective effects of GOx@ZT-MN on cells through ROS clearance, H_2_O_2_ and each group of microneedles were co-cultured with cells along with a PBS solution. This experimental setup allowed for dynamic monitoring of mitochondrial function. Mitochondria are sensitive indicators of cell damage, and a decrease in their membrane potential (MMP) is an early indicator of apoptosis. Normal mitochondria, due to their high membrane potential, induce JC-1 to form red fluorescent polymers. In comparison, apoptotic cells, due to membrane potential depolarization, cause JC-1 to depolymerize into green fluorescent monomers. As presented in Fig. 6C, the proportion of green fluorescence density in the H_2_O_2_ and H_2_O_2_+MN groups increased significantly and indicated significant

mitochondrial dysfunction. The fluorescence distribution of the GOx@ZT-MN treatment group, however, was similar to that of the normal control group, thus confirming its effectiveness in maintaining mitochondrial membrane potential of fibroblasts while clearing ROS. Moreover, live/dead cell double staining experiments indicated a significant improvement in the cell survival rate of the GOx@ZT-MN group (92.58 ± 1.31 %) and GOx@ZT group (93.89 ± 1.11 %) treated group compared to the H_2_0_2_ group (26.18 ± 1.58 %) (Fig. 6B and E). This verifies, from the perspectives of both cell metabolism and structural integrity, that this system protects fibroblasts by regulating the ROS-mitochondrial axis and its associated molecular mechanisms of apoptosis pathways. This multifaceted protective effect significantly enhances cell survival.

### 2.9 GOx@ZT-MN's ability to promote cell proliferation, migration and angiogenesis *in vitro*

**Fig. 7:**
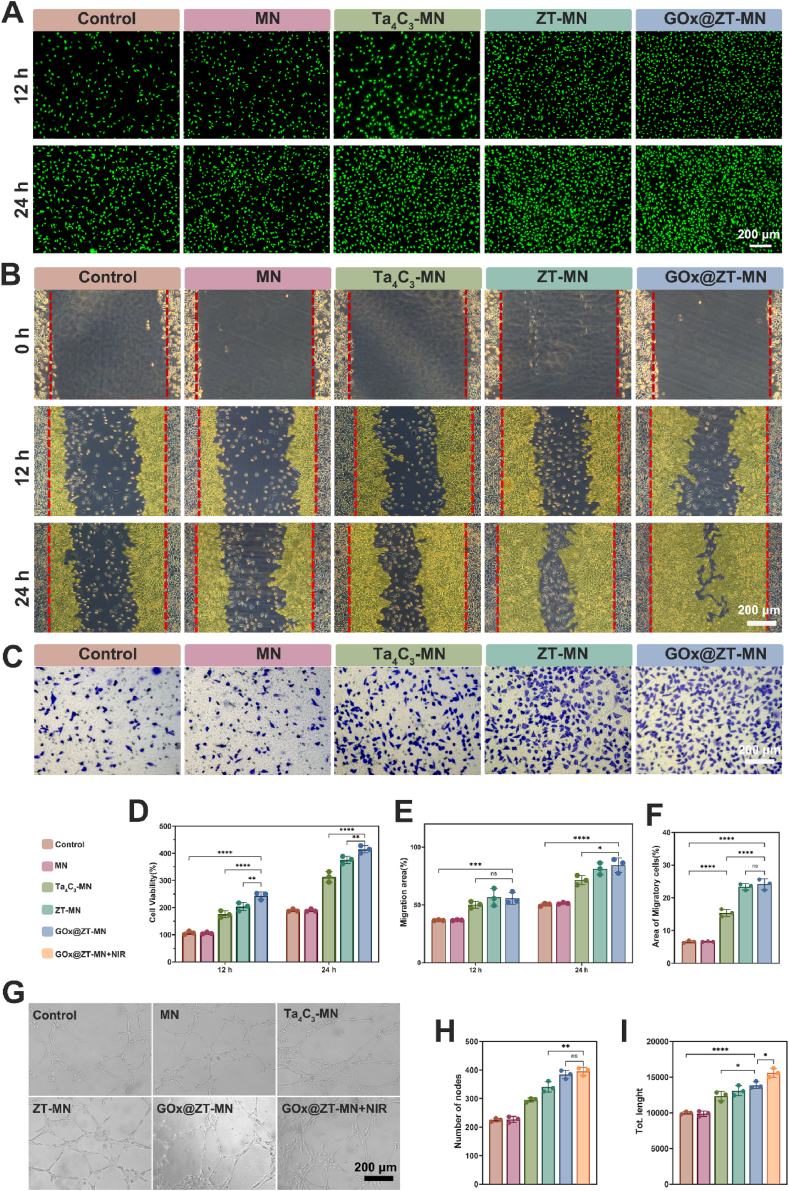
Biological effects of GOx@ZT-MN *in vitro*. (A) Calcein-AM (green fluorescence) staining of L929 cells after immersion with microneedles and its (D) quantitative analysis of Cell viability. (Scale bar = 200 μm) (B) Optical images of the scratch assays of L929 cells after immersion with microneedles and its (E) quantitative analysis of Migration area. (Scale bar = 200 μm) (C) Transwell optical images of L929 cells migration and its (F) quantitative analysis of Migration area. (Scale bar = 200 μm) (G) Representative optical images of tube formation assay of HUVECs after immersion with microneedles and its quantitative analysis of (H) nodes and (I) length. Data are presented as mean ± SD (n = 3). (Scale bar = 200 μm) ∗*P* < 0.05, ∗∗*P* < 0.01, ∗∗∗*P* < 0.001, ∗∗∗∗*P* < 0.0001. ns: no significant difference. (For interpretation of the references to color in this figure legend, the reader is referred to the Web version of this article.)

In addition to ROS elimination, fibroblast proliferation and migration, along with angiogenesis, are critical markers of diabetic wound healing [70]. We performed proliferation and migration assays utilizing mouse fibroblast (L929) cells and tube formation assays with HUVECs. Proliferation assays indicated that L929 cells exhibited notable proliferative capacity after 12 h of incubation, and these cells were cultured at uniform density with different microneedles, particularly in the presence of GOx@ZT-MN. L929 cell counts reached levels similar to the control group (243.80 ± 11.08 %), with the ZT-MN group (203.55 ± 12.22 %) demonstrating similar behavior. The Ta_4_C_3_-MN (175.47 ± 10.24 %) group also outperformed the control group (Fig. 7A and D), while the MN group demonstrated no significant difference from the control. At the 24-h time point, the GOx@ZT-MN group exhibited a fourfold increase in density compared to the 12-h measurement. This increase suggests synergistic effects among the GOx@ZT-MN components that stimulate cell growth. Cell migratory capacity represents another key indicator of cellular function, and L929 cells were adhered for a scratch assay that aimed to assess the effect of each microneedle group on L929 cell migration (Fig. 7B and E). The GOx@ZT-MN group exhibited robust migratory capacity (55.71 ± 4.13 %) and occurred after a 12-h scratch assay. At 24 h post-scratching, L929 cell migration in the GOx@ZT-MN group was further enhanced (84.30 ± 4.13 %). The ZT-MN (81.26 ± 4.15 %)

and Ta_4_C_3_-MN (71.40 ± 3.34 %) groups also demonstrated a significantly greater ability to promote cell migration compared to the control (50.30 ± 1.01 %) and MN (51.41 ± 0.58 %) groups. These results indicate that GOx@ZT-MN facilitates cell growth toward the wound site and has a good ability for promoting cell healing. A Transwell cell migration assay further corroborated the finding that each microneedle component significantly enhanced fibroblast migration. After 24 h of culture, minimal cell recruitment to the lower chamber was observed in the control (6.59 ± 0.29 %) and MN (6.31 ± 0.92 %) groups; whereas, significantly more cells were observed in the ZT-MN (23.37 ± 0.77 %) and Ta_4_C_3_-MN (15.40 ± 0.90 %) groups compared to the control group (6.59 ± 0.29 %). The highest cell count in the field of view was observed in the GOx@ZT-MN group (24.14 ± 1.35 %) and was indicative of a potent cell recruitment capacity ((Fig. 7C and F). In *vitro* Matrigel angiogenesis assays were performed to assess the pro-angiogenic potential of the microneedles. The number of nodes is the highest in the GOx@ZT-MN (384.33 ± 11.90 %) group and GOx@ZT-MN + NIR group (395.67 ± 11.26 %) and form a dense vascular network. The number of vascular nodes per unit area of Matrigel surpassed 350 in both microneedle groups, and vessel length was also significantly better than that of the other four groups. This effect may be related to the mild photothermal effect and the increased temperature results in more release of Zn^2+^ and H_2_S; they synergistically enhance angiogenesis, consistent with their role in promoting cell proliferation [71] (Fig. 7G, H and 7I). In summary, these experimental results demonstrate that GOx@ZT-MN effectively promotes cell proliferation, migration, and angiogenesis.

### 2.10 GOx@ZT-MN *in vitro* tests to promote wound healing in diabetic wounds

**Fig. 8:**
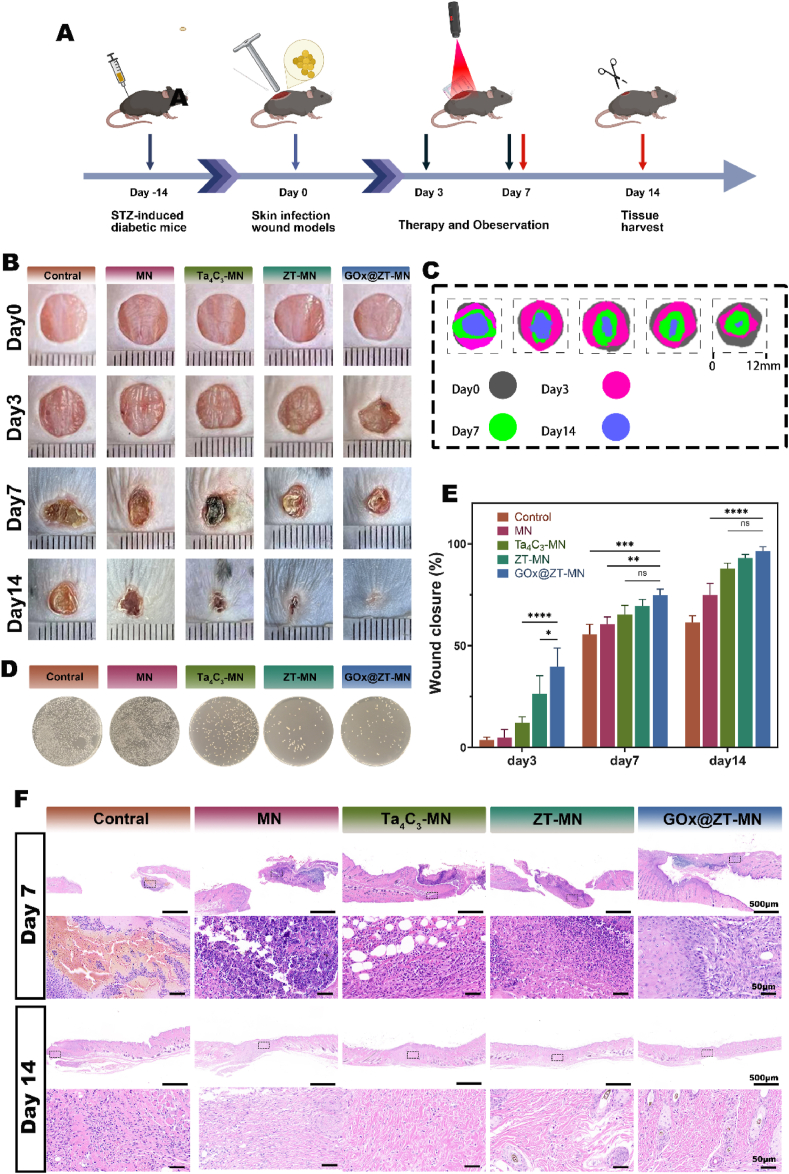
GOx@ZT-MN accelerates wound healing in MRSA-infected type Ⅱ diabetic C57 mice *in vivo*. (A) Schematic illustration of the modeling and treatment process. (B)Photos depicting the wound healing progression on postoperative days 3, 7, and 14. (Width = 12 mm) (C) Diagrammatic representation of changes in wound area during the healing process. (D) Optical photographs of MRSA samples taken from mice for culture. (E) Quantitative analysis of wound closure rates on days 3, 7, and 14 post-surgery. (F) Images of H&E -stained wounds at 7 and 14 days after different microneedles treatments. (Scale bar = 500 μm, 50 μm). Data are presented as mean ± SD (n = 3). (Scale bar = 200 μm) ∗*P* < 0.05, ∗∗*P* < 0.01, ∗∗∗*P* < 0.001, ∗∗∗∗*P* < 0.0001. ns: no significant difference.

**Fig. 9:**
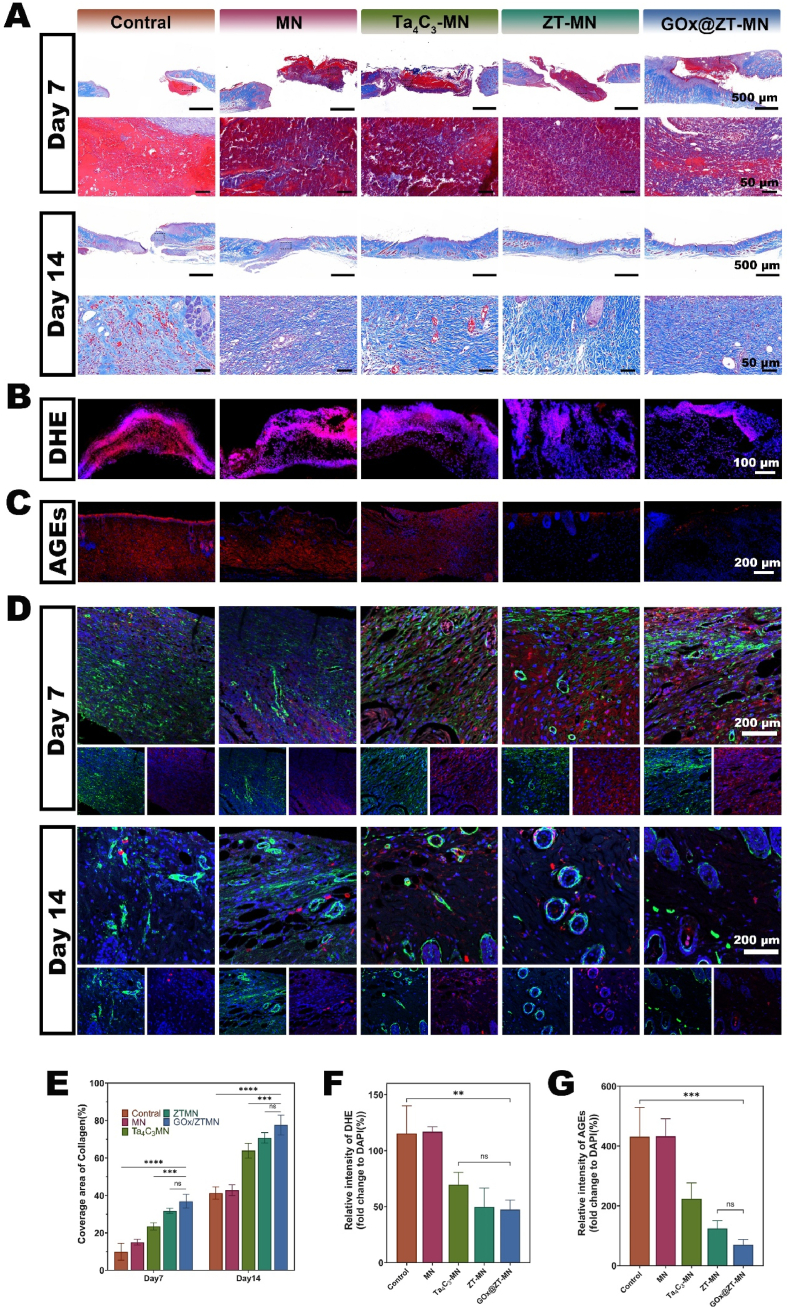
Histological analysis of MRSA infected diabetic wound. (A) Images of masson trichrome staining of wound sections on postoperative days 7 and 14. (Scale bar = 500 μm, 50 μm). (B) Images and (F) quantitative analysis of DHE fluorescence staining. (Scale bar = 100 μm). (C) Images and (G) quantitative analysis of AGEs fluorescence staining. (Scale bar = 200 μm). (D) Images of CD31/αSMA immunofluorescence staining. (Scale bar = 200 μm) (E) Quantitative analysis of collagen deposition in an infected diabetic mouse model at different time periods after Control, MN, Ta_4_C_3_-MN, ZT-MN and GOx@ZT-MN groups treatments. Data are presented as mean ± SD (n = 3). (Scale bar = 200 μm) ∗*P* < 0.05, ∗∗*P* < 0.01, ∗∗∗*P* < 0.001, ∗∗∗∗*P* < 0.0001. ns: no significant difference.

GOx@ZT-MN exhibits excellent antibacterial properties, effectively clears ROS, and promotes cell proliferation, migration, and angiogenesis. This suggests that it has considerable potential for treating infected diabetic wounds. To thoroughly evaluate this therapeutic potential, a murine model of type II diabetes was established, and on this basis, an 8 mm cutaneous infection defect model was generated. Five treatment groups were implemented. Each group employed different microneedle formulations or physiological saline. Treatment regimens consisted of daily near-infrared light irradiation (1.5Wcm^−2,^ 808 mm, 5min) and microneedle

replacement (Fig. 8A). Photographic and quantitative assessments of wound closure are presented in Fig. 8B and D. By day 7, the majority of wounds in the GOx@ZT-MN, ZT-MN, and Ta_4_C_3_-MN groups had closed (74.86 ± 2.36 %). This closure demonstrated significantly improved healing compared to the control (55.57 ± 3.98 %) and MN (60.50 ± 2.96 %) groups. By day 14, wound closure in the GOx@ZT-MN group was nearly complete (96.41 ± 1.72 %) (Fig. 8C). This represented a significantly enhanced healing response compared to the other four groups. Following NIR irradiation, the ZT-MN, Ta_4_C_3_-MN, and MN groups demonstrated near-complete wound closure. Of particular note, the healing rate observed in the MN group was significantly greater compared to that of the control group. We speculated that this enhanced healing may be attributed to the mechanical stimulation induced by the microneedles. Further analysis was conducted. This involved the cultivation of bacteria obtained from the wound surface after a 24-h period, which indicated a dense coverage of MRSA colonies in both the MN and control groups, with colony counts in excess of 1500 (Fig. S13). The GOx@ZT-MN and ZT-MN treatments exhibited the most antibacterial effect, resulting in a near-complete eradication of the bacterial population. The Ta_4_C_3_-MN treatment was not as effective in fully eliminating MRSA, when compared to the GOx@ZT-MN and ZT-MN interventions (Fig. 8D). Then, histological analysis of wound sections was performed. This followed sectioning and staining procedures and offered further insights. Hematoxylin and eosin HE staining indicated the presence of blood staining in the wound tissues of both the control and MN groups at the one-week time point. These groups also displayed significantly impaired healing, reflected by separated skin tissue and the content of inflammatory factors was the highest. In contrast, the GOx@ZT-MN group demonstrated the best healing effect. This was evident as they exhibited essentially complete re-epithelialization while producing stable skin structures by the second week of observation. The control group, at this same time point, demonstrated incomplete tissue regeneration and no stable epidermal structures were established. Masson's trichrome staining produced similar observations (Fig. 8F). At the one-week time point, both the control and MN groups exhibited persistent blood stains. They also demonstrated a near absence of collagen deposition; whereas, the GOx@ZT-MN group demonstrated the largest collagen deposition (36.90 ± 2.99 %), which was in sharp contrast to the limited collagen deposition observed in the control group (15 ± 1.32 %). By the second week, the GOx@ZT-MN group displayed evidence of significantly formed well-defined collagen fibers (Fig. 9A and E).

In order to further understand the efficiency of ROS clearance in diabetic patients with wound healing and the effect of gel on the progression of inflammation, we conducted DHE immunofluorescence staining on wound tissue 3 days after surgery. The fluorescence of GOx@ZT-MN, ZT-MN and Ta_4_C_3_-MN was significantly reduced (Fig. 9B and F), which is inseparable from the ability of MXenes to eliminate ROS across a broad spectrum. Advanced glycation end products (AGEs) are typically harmful metabolic products in a hyperglycemic environment, and their accumulation leads to increased inflammation and oxidative stress. We measured AGE levels in wound tissue after treatment in diabetic mice. Compared with other groups, AGE levels in GOx@ZT-MN group were significantly reduced (Fig. 9C and F). This reduction can be attributed to GOx@-ZT-MN's role in lowering local blood sugar and regulating wound inflammation, thereby minimizing the formation and accumulation of AGEs.

The analysis of CD31 and α-SMA indicated higher fluorescence densities for GOx@ZT-MN, ZT-MN, and Ta_4_C_3_-MN compared to the MN and Control groups during the first week. This suggests a strong pro-angiogenic effect for GOx@ZT-MN, ZT-MN, and Ta_4_C_3_-MN. By

week two, fluorescence densities for GOx@ZT-MN, ZT-MN, and Ta_4_C_3_-MN decreased, whereas those for the control and MN groups gradually rose. This delayed angiogenic and healing response in the control and MN groups indicates early vascular development. In the second week, increasing tube diameter and decreasing angiogenesis in the GOx@ZT-MN group suggest mature vessel formation, whereas the control and MN groups remained in the proliferative vascular phase (Fig. 9D). This further supports a delay in angiogenesis in the control and MN groups, with GOx@ZT-MN significantly expediting the process. Considering the critical role of vascularization in wound tissue regeneration, these data indicate that GOx@ZT-MN significantly promotes wound healing. In addition to tissue repair and antibacterial action, macrophage-mediated immunomodulation is essential.

**Fig. 10:**
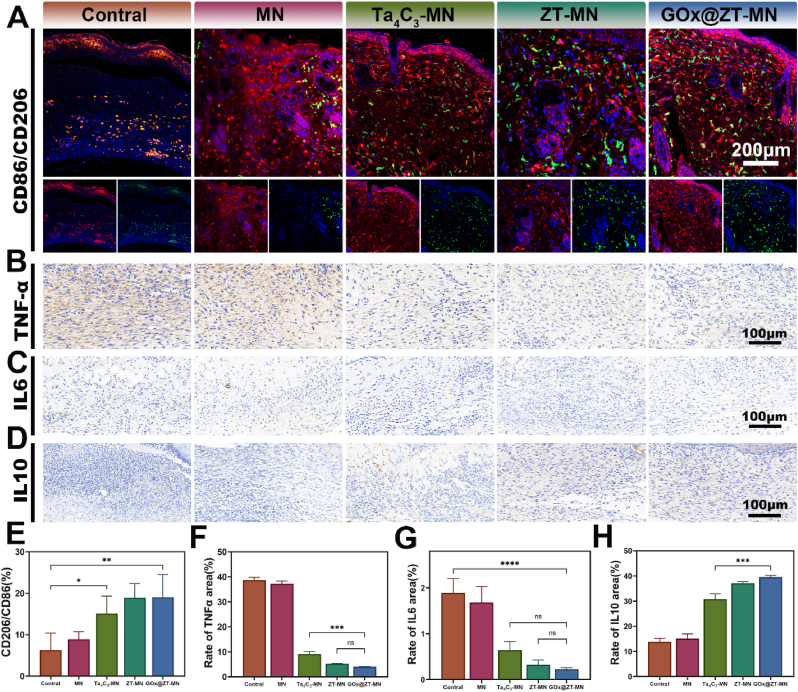
Histological analysis of MRSA infected diabetic wound. (A) Images and (E) quantitative analysis of CD206/CD86 immunofluorescence staining. (Scale bar = 200 μm) (B–D) Immunohistochemical images and (F–H) quantitative analysis of TNF-α, IL6 and IL10. (Scale bar = 100 μm) Data are presented as mean ± SD (n = 3). ∗P < 0.05, ∗∗P < 0.01, ∗∗∗P < 0.001, ∗∗∗∗P < 0.0001. ns: no significant difference.

Immunofluorescence staining for CD206 and CD86 on diabetic infected wound sections indicated a predominance of anti-inflammatory M2 macrophages in the GOx@ZT-MN group in one week. This was followed by the ZT-MN, Ta_4_C_3_-MN, MN, and Control groups, displaying decreasing levels of CD206; whereas, CD86, a marker for M1 macrophages, demonstrated the opposite fluorescence pattern (Fig. 10A and E). To further explore the effect of microneedles on the inflammatory response, immunohistochemical analysis of the pro-inflammatory cytokines IL-6 and TNF-α, as well as the anti-inflammatory cytokine IL-10, was performed. GOx@ZT-MN exhibited the highest IL-10 expression, followed by ZT-MN, Ta_4_C_3_-MN, MN, and finally the Control group. The highest expression of IL-6 and TNF-α was observed in the control group, followed by the groups in reverse order of IL-10 expression (Fig. 10B–D, 10F-H). These findings suggest that GOx@ZT-MN reduces inflammatory responses, in part, through the modulation of macrophage polarization.

**Fig. 11:**
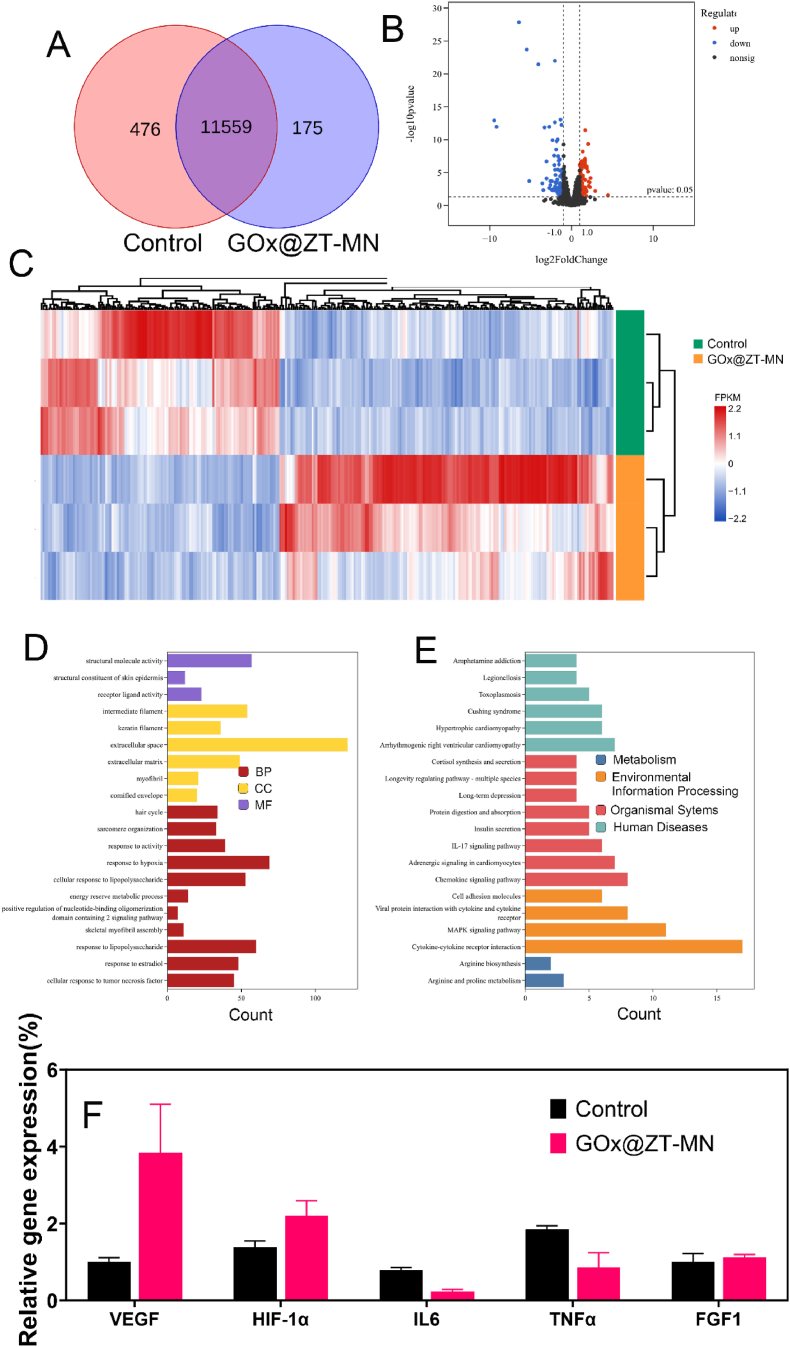
RNA sequencing analysis of GOx@ZT-MN versus control mice after 7 days of treatment. (A) Venn diagram and (B) volcano of the number of expressed genes in tissue after treatment with GOx@ZT-MN and controls. (C) Heatmap analysis of transcriptional expression profiles after GOx@ZT-MN. (D) GO enrichment analysis elucidates the involvement of cellular components, molecular functions, and differential gene expression in various biological processes. (E) KEGG pathway enrichment analysis provides insights into the pathways associated with the differentially expressed genes. (F) Relative expression levels of HIF-1α, VEGF, IL6, TNFα, and FGF1 were measured by real-time fluorescence-equivalent q-PCR (n = 3). Data are presented as mean ± SD (n = 3).

To elucidate the molecular mechanisms underlying GOx@ZT-MN-promoted infectious wound healing in diabetic mice, skin tissues from the GOx@ZT-MN treatment group and control group (n = 3) were collected on day 7 post-modeling for RNA sequencing analysis. Full transcriptome sequencing detected a total of 12210 genes, of which 476 genes were unique to the Control group and 175 genes were unique to the GOx@ZT-MN treated mice. with differential expression analysis (Control vs GOx@ZT-MN) identifying 166 significantly up-regulated genes and 199 significantly down-regulated genes (Fig. 11A and B). Box plot results show that GOx@ZT-MN has good gene abundance (Fig. S14), hierarchical clustering-based heat maps revealed distinct transcriptomic profiles in the GOx@ZT-MN group (Fig. 11C). To further investigate differences in Molecular Function (MF), Cellular Composition (CC), Biological Processes (BP), and associated pathways, we performed Gene Ontology (GO) and Kyoto Encyclopedia of Genes and Genomes (KEGG) enrichment analyses. The results demonstrated significant disparities between the two groups in genes and pathways related to immune-inflammatory

response, oxygenation response, energy metabolism, tissue regeneration, and insulin signaling (Fig. 11D and E). A deeper examination of differentially expressed genes showed that GO enrichment analysis identified the top 20 most significantly upregulated genes in the GOx@ZT-MN group as being associated with tissue regeneration (e.g., collagen fibers and muscle tissue), indicating a transition from the inflammatory phase to the repair phase in diabetic wounds treated with GOx@ZT-MN. Conversely, the top 20 most significantly downregulated genes were primarily linked to immune-inflammatory response genes. KEGG enrichment analysis yielded consistent findings (Fig. S15). These results are consistent with our previous experimental outcomes, confirming that GOx@ZT-MN effectively mitigates the inflammatory response at the wound site and accelerates wound healing. Additionally, GOx@ZT-MN enhances tissue healing by promoting oxygenation, which provides an additional therapeutic benefit. We employed q-PCR to evaluate the expression levels of genes associated with dermal wound oxygenation, specific inflammatory responses, and angiogenesis. On day7, HIF-1α levels were significantly elevated compared to the control group, likely due to the enzymatic reaction of GOx generating a substantial amount of oxygen. Moreover, GOx@ZT-MN downregulated inflammation-related genes (IL6 and TNFα) while upregulating those involved in wound repair genes (VEGF and FGF1) (Fig. 11F).

## 3 Conclusion

This study offers an overview of key factors contributing to infectious diabetic wounds and presents a targeted treatment approach. A "three uses in one sheet" of Ta_4_C_3_ MXenes platform was developed, incorporating high surface area, in situ grown ZnS nanoparticles, and photothermal antibacterial agents to stimulate blood vessel formation and remove ROS in the wound. This GOx@ZT-MN construct combines zinc sulfide (ZnS) nanoparticles with Ta_4_C_3_ MXenes nanosheets, loaded onto microneedles along with GOx. Microneedles act as a delivery carrier and offer advantageous solubility, adhesion, mechanical strength, and biocompatibility. This allows for a multi-modal, synergistic treatment of infected diabetic wounds that combines gas and photothermal therapies. This strategy protects the wound tissue by regulating the ROS-mitochondrial axis to prevent cells from apoptosis, and uses a synergistic photothermal gas-ion mechanism to be antibacterial and anti-inflammatory. Moreover, it encourages cell proliferation, migration, and angiogenesis. It also promotes collagen deposition and modulates macrophage polarization, which reduces inflammation. This multi-module treatment strategy offers a novel approach to managing diabetic wounds infected with drug-resistant bacteria. However, it should be noted that the current development cost of the core material Ta_4_C_3_ MXene is high, and there are inconsistent quality controls in the mold manufacturing process. Future research can focus on optimizing the MXene synthesis process, while exploring bioprinting technology for precise printing, further promoting the transformation of this treatment strategy from laboratory to clinical application, and making it applicable to the field of diabetic wound treatment and even other acute and chronic wounds.

## CRediT authorship contribution statement

**Shihao Deng:** Writing – review & editing, Writing – original draft, Methodology, Conceptualization. **Yunhao Tai:** Resources, Methodology. **Chenxu Liu:** Resources, Methodology. **Kenan Sun:** Methodology. **Shaoze Lan:** Methodology. **Liu Yang:** Methodology. **Canming Ye:** Methodology, Conceptualization. **Li Huang:** Writing – review & editing. **Runhuai Yang:** Methodology. **Haisheng Qian:** Methodology. **Jun Li:** Writing – review & editing, Funding acquisition, Conceptualization.

## Ethics approval and consent to participate

Not applicable.

## Ethics approval statement

The animal study was approved by the Animal Ethics Committee of the authors’ institution (LLSC 20242390). All experimental steps were approved by the Committee on the Ethics of Animal Experiments of Anhui Medical University.

## Consent for publication

Not applicable.

## Funding

This study was financially supported by Natural Science Foundation of Hefei City (No. 2022041), Clinical Research cultivation Program of the Second Affiliated Hospital of Anhui Medical University (No. 2020LCZD20), Research Fund of Anhui Institute of translational medicine (No. 2022zhyx-C44), and Basic and Clinical Cooperative Research Promotion Plan of Anhui Medical University (No. 2020xkjT040)S, Tibet Autonomous Region Natural Science Foundation Grouped Assistance to Tibet Medical Project (No. XZ2023ZR-ZY47(Z)), Provincial Quality Engineering Program for Higher Education Institutions in Anhui Province (No. 2022sx075 and No. 2022jyxm739), and The New Era Education Quality Project of Anhui Provincial Department of Education (2023zyxwjxalk054).

## Declaration of competing interest

The authors declare that they have no known competing financial interests or personal relationships that could have appeared to influence the work reported in this paper.

## Data Availability

Data will be made available on request.
