# Supplementary material for: Multifunctional microneedle-mediated photothermo-gas-ion synergic therapy accelerates MRSA infacted diabetic wound healing
Source: Mater Today Bio. 2025 May 24;32:101903. doi: 10.1016/j.mtbio.2025.101903 (PMC12162066; doi:10.1016/j.mtbio.2025.101903)
Supplement: Multimedia component 1 [file mmc1.docx]

## Support materials and methods

### Materials

MAX (Ta_4_AlC_3_) was purchased from Jiangsu Xianfeng Technology Co., Ltd., and reagents for HFsolution, PEGDA, acrylamide (AM), glucose oxidase (GOx), DCFH-DA kit, PEG3000, streptozotocin and methacrylate gelatin were purchased from Aladdin Reagent Company, Ltd. (Shanghai, China). The microneedle mold was purchased from Guangzhou Enming Biotechnology Co., Ltd. (China). thiourea,[Zn(CH_3_COO)_2_]·2H_2_O, Tetrapropylammonium hydroxide (TPAOH) was purchased from McLean Biochemical Technology Limited (Shanghai, China). Fetal bovine serum was purchased from Thermo Company (USA). 24 cross-chamber well plates, 4% paraformaldehyde, and crystal violet solution were purchased from Wuhan Sevier Biotechnology Company (China). Matrigel Matrigel, various wells, ordinary culture dishes, and confocal culture dishes were purchased from Corning Company (USA), CCK-8 reagent was purchased from Biosharp Company (USA), and Cyan/Streptomycin was purchased from Beijing Solaibao Technology Co., Ltd. DMAO/PI bacterial stain and live/dead cell stain were purchased from Shanghai Beibo Biotechnology Co., Ltd. DMEM high-sugar medium and pancreatin cell digestion fluid were purchased from Gibco Company (USA). PBS buffer was purchased from Hyclone Company (USA).

Methoxy-resistant Staphylococcus aureus were purchased from the Institute of General Health of Hefei National Science Center, L929 cells and human umbilical vein endothelial cells (HUVECs) cell lines were purchased from Fuheng Biotech, and DMEM medium containing 10% FBS and 1% penicillin-streptomycin was used in vitro. Cultivate at 37°C and 5% CO_2_. The C57 mice used in the in vivo experiments were from the Animal Experiment Center of Anhui Medical University.

### Synthesis and method of GOx@ZT-MN

#### 2.1 Synthesis of single layer MXene

Firstly, the Al elements of multilayer MAX(Ta_4_AlC_3_) were removed by etching in 50% HF solution to obtain multilayer MXene(Ta_4_C_3_) like shingles stacked, and then the prepared multilayer MXene(Ta_4_C_3_) was put into Tetrapropylammonium hydroxide (Tetrapropylammonium hydroxide_3_, TPAOH) were intercalated to produce single-layer MXene(Ta_4_C_3_) nanosheets.

#### 2.2 Synthesis of ZT nanoplatform

The ZnS/Ta_4_C_3_, or (ZT) nanoplatform, was generated in situ by a hydrothermal method. A single-layer Ta_4_C_3_ nanoplate was heated with [Zn (CH_3_COO)_2_]·2H_2_O and thiourea in an autoclave at 140℃ for 12 hours, and finally washed, centrifuged and dried to obtain the ZT nanoplatform

#### 2.3 Synthesis of GOx@ZT

GOx@ZT was synthesized through amidation reaction. 10mg of GOx and 20mg of ZT nanoplatforms were dispersed in 10ml of PBS and stirred magnetically for 24 hours. During this period 40mg of carbodiimide hydrochloride and 60mg N-hydroxysuccinimide is combined with the ZT nanoplatform. Finally, the obtained mixture was centrifuged (13,000 rpmmin^−1^, 10mins) and washed with PBS, and finally, free GOx was removed by dialysis (100KDa MW). The resulting nanoparticles were stored at 4℃.

#### 2.4 Synthesis of GOx@ZT-MN

The microneedle patch is manufactured by a secondary photo-curing method. The tip of the microneedle is made of mixed photo-curing of PEGDA and PEG_3000_ solutions and GOx@ZT particles, and the backing layer is made of a combination of adhesive acrylamide and PEGDA. According to the subsequent photo-thermal conversion efficiency results,

GOx@ZT with a concentration of 400μg mL^-1^ was finally selected to form the microneedle tip for subsequent experiments. According to the microneedle manufacturing plan of the intestinal robot, that is, a mixed aqueous solution of 33% by weight PEG_3000_, 33% by volume PEGDA, 0.33% by weight photoinitiator 819, and 0.033% by weight dye Sudan Ⅰ was poured onto the PDMS microneedle mold, and vacuum was applied. The cotton swab absorbed part of the liquid on the backing layer of the mold, and the volume ratio of PEGDA and AM was half. A mixture of 0.1% by weight of Lap photoinitiator was placed on the backing layer of the mold, then photocured again for 30 seconds, dried for 30 minutes, and finally GOx@ZT-MN was obtained through mold release. The rest of the microneedles were subsequently synthesized by using the same mole of MXene(Ta_4_C_3_) and the ZT nanoplatform.

### 3. Characterization of GOx/ZTMN

#### 3.1 Scanning electron microscope (SEM)

Take the prepared multilayer MAX(Ta_4_C_3_), single-layer MXene(Ta_4_C_3_), ZT nanoplatform, pure PEGDA and GOx@ZT-MN and place them into the gold spraying chamber for gold spraying to prevent affecting the shooting effect. Then use conductive glue to stick it to the sample table, evacuate the air to vacuum, adjust the instrument voltage, adjust the height of the sample table, find the sample at the required multiple, and scan the image. Select a good field of view under the mirror in the sample, test the morphology on one side of the sample and the energy spectra of C, Ta, Zn, S and other elements, and then select a line area in the appropriate direction for scanning and testing. After all tests are over, save the exported images and data.

#### 3.2 Transmission electron microscope (TEM)

Multi-layer MAX(Ta_4_C_3_), single-layer MXene(Ta_4_C_3_), and ZT nanoplatforms were fabricated on copper networks. Place the sample on the sample stage at the head of the sample rod, insert the equipment, and adjust to the appropriate brightness. First find a specific position in TEM mode, then switch the shooting mode to STEM mode, adjust parameters such as electron beam, aperture, focal length, astigmatism, etc., and adjust to the appropriate brightness for shooting.

#### 3.3 X-ray diffraction (XRD)

Remove excess water from the ZT nanoplatform and GOx@ZT-MN, and place the sample to be tested on the sample tray. Send the prepared sample to the sample introduction chamber to withdraw vacuum, and send the sample to the analysis chamber. Test collect data, save and export the data.

#### 3.4 Fourier Transform Infrared Spectroscopy (FTIR)

Take out the freeze-dried sample prepared above, place the ZT nanoplatform and GOx@ZT-MN in the optical path of the infrared spectrometer (Thermo Scientific Nicolet), scan the air background, and then place the surface to be measured of the sample tightly against the infrared transparent crystal surface of the ATR attachment. Scan the infrared spectrum of the surface to be measured of the microneedle sample, with a wave number range of 400-4000cm^-1^, and make a curve graph with the wave number range as the abscissa.

#### 3.5 Zeta potential

The potential of GOx@ZT-MN and ZT powders was tested using a nanometer particle size and Zeta potential analyzer (Malvern, UK, Zetasizer NanoZS 90) with deionized water as a dispersant, and the potential of GOx@ZT-MN and ZT powders was tested using GraphPadPrism10 performs statistical plots.

#### 3.6 Skin penetration test

For the microneedle compression test, an electronic universal testing machine (INSTRON, USA) was used to test, and the microneedle patch was adhered to a stainless steel moving table and positioned facing force. The stage moves at a rate of 0.1mm s^-1^, and when the microneedle contacts the force-measuring chronograph, the relationship between force and displacement is measured. To assess further skin penetration of GOx@ZT-MN in vitro, GOx@ZT-MN was pressed into the dorsal skin of mice for 5 minutes, the MN was removed, the skin was fixed in 4% paraformaldehyde, the fixed skin tissue was embedded in paraffin and sections, and Hematoxylin and Eosin (HE) staining was performed.

### 4. Performance testing of GOx@ZT-MN

#### 4.1 Photothermal performance

Put 100μgml^-1^, 200μgml^-1^, 400μgml^-1^, and 800μgml^-1^ in PBS solutions into an EP tube containing 1 ml of PBS solution, and use a near-infrared device (Fuzhe Light, FU808AD3000-F100) to irradiate them with a near-infrared light of 808nm at a power of 1.5Wcm^−2^. A fixing frame was used to fix the thermal imager (HIKMICRO, TPK10) 20cm away from the sample and monitor the temperature of the EP tube, and take photos and record every minute. Taking the highest temperature of the EP tube core at each time point as the ordinate and time as the abscissa, a temperature change curve is drawn. Then, the test will be carried out according to the above steps using 808nm infrared light of 1.0Wcm^−2^ and 0.5Wcm^−2^ respectively. Place GOx@ZT-MN in a humid environment, use a thermal imager to monitor the temperature of the microneedle every minute, repeat the switch test three times, and take photos to record.

#### 4.2 DPPH radical scavenging ability

Use an analytical balance to accurately weigh 20.0mg of DPPH (1,1-diphenyl-2-trinitrophenylhydrazine) standard, fully dissolve it in 60ml of anhydrous methanol, and place it in a brown reagent bottle at 4°C as the reaction solution. Soak equal amounts of reaction solution and GOx@ZTMN for 1, 3, 6, 12, and 24 hours respectively, observe the color change of the reaction, and aspirate the liquid into a 96-well plate. Using a microplate reader, the absorbance values of each group of solutions were measured at the wavelength of 517nm.

DPPH clearance (%)=(A_0_-A_1_)/A_0_*100%(A_1_: determination group; A_0_: blank group), where the blank group is an equal volume of deionized water instead of the sample solution.

#### 4.3 EPR EPR spin-trapping experiment ^-^

5,5-dimethyl-1-pyrroline-n-oxide (DMPO) was applied as a spin trap to enable the identification of radicals as long-lived DMPO-radical adducts, reacted in dark light for 2 min, and the signal was captured.

#### 4.4 Enzyme activity testing of GOx

Prepare 10ml of a 25mM glucose solution, place GOx@ZT nanoparticles (1mg), the same mole of GOx(308mg) and GOx@ZTMN (needle tip portion) at room temperature (22℃) and in a water bath at 50℃ respectively for reactions, collect the reacted solutions at different time points, and measure them using a blood glucose meter, where the enzyme activity is equal to (C_0_-C_1_)/C0 *100%, C_1_: the blood sugar concentration at different reaction times, and C_0_: the initial concentration of the reaction. The DMEM cell medium was added with glucose to high glucose state (≈17mmol/L), and then co-cultured with microneedles. The glucose concentration in the medium was measured by glucometer, and AM staining was performed at 24 hours. After the microneedles were gently attached to the wound, the wound exudate was collected at 6, 12, 24 and 48 hours for glucose concentration test.

#### 4.5 Release of H_2_S and Zn^2+^

GOx@ZTMN was immersed in various PBS solutions (pH=7.4, 6.2, 5.0) for different periods of time (1, 2, 4, 6, 9, 12, and 24 hours), placed on a thermostatic shaker at 37°C, filtered, and H2S release was determined according to the standard methylene blue method. In short, add 1ml of a mixed solution of zinc acetate and sodium acetate (mass ratio=4:1). The precipitate was collected by centrifugation (13000rpm, 40minutes) and re-dissolved with FeCl_3_ (5mg/ml) solution and mixed with N, N-dimethyl-p-phenylenediamine sulfate. After incubation at 37°C for 15 minutes, methylene blue was formed and the absorbance was measured at 665nm. For Zn^2+^, it was measured using inductively coupled plasma-mass spectrometry (ICP-MS), and release curves were prepared on GraphPadPrism10 based on the above data.

### 5 Biocompatibility of GOx@ZT-MN

#### 5.1 blood compatibility

Eye blood from C57 mice was collected in anticoagulant tubes containing sodium citrate. After centrifugation at 3000rpm for 15 minutes, 20μl of underlying blood cells were extracted and mixed with the following solutions: 1ml of deionized water (DW), 1ml of PBS, and 4bottles of 1ml of PBS solution were mixed with each group of microneedle materials. The mixture was incubated at 37℃ for 4 hours and centrifuged at 3000rpm for 15 minutes. Subsequently, 100μl of supernatant was transferred to a 96-well plate, and the OD value of each group's supernatant at 545nm was determined by a microplate reader. The OD value of the positive control group (deionized water) was recorded as 100% hemolysis, and the negative control group (PBS) was marked as 0% hemolysis. Hemolysis rate =( OD_B_-OD_A_)/( OD_C_-OD_A_) ×100%(OD_A_; PBS group; OD_B_: sample group; OD_C_: pure water group).

### 5.2 CCK-8 Experiment

Log phase mouse fibroblast (L929) and human umbilical vein endothelial cells (HUVECs) cells were seeded into 96-well plates at a density of 2×10^4^/ml. Four experimental groups and a control group were set up, namely MN group, Ta_4_C_3_-MN group, ZT-MN group, GOx@ZT-MN group and control group. After the cells in the cell suspension adhered to the wall (about 3 hours), microneedles were added to each group, and three 96-well plates were cultured at 37℃ and 5% CO_2_ for 1 day. Remove the well plate, remove the culture medium and placed microneedles, add 200μl of DMEM medium containing 10% CCK-8 to each well, incubate in a cell incubator protected from light for 4 hours, and use a microplate reader (Varioskan, LUX) to detect the absorbance at 450nm. Cell survival rate (%)= A_1_/A_2_*100% (A_1_: experiment group, A_2_: control).

#### 5.3 in vivo compatibility

In order to avoid the impact of the type Ⅱ diabetes model on major organs such as liver, liver, spleen, lung and kidney, we used healthy C57 mice and divided them into blank group, MN group, Ta_4_C_3_-MN group, ZT-MN group and GOx@ZT-MN group according to material groups. Each group had 3 mice. A full-thickness skin wound model with a diameter of 8mm was established on the back of the mice, and treated with microneedles in each group. Each group was irradiated with 808nm infrared light with a power of 1.5Wcm^−2^ for 5 minutes every day. Half a month later, the mice were euthanized. The liver, liver, spleen, lung, kidney and viscera of mice were taken out for pathological sections and HE staining.

### 6 In vitro antibacterial ability of GOx@ZT-MN

#### 6.1 Plate count

Take 1×10^7^cfu/mL bacterial solution and dilute it 10000 times, evenly spread 200μl of the diluted bacterial solution on LB solid medium, divide it into 5 groups, with 3 samples in each group, and co-cultivate it with different microneedle extractions for 1 day. The other 5 groups were the same as the above group, but were subjected to NIR irradiation (1.5Wcm^-2^, 808nm, 5min), photographed the next day and colony counting was performed with ImageJ.

#### 6.2 Crystal violet staining biomembrane

Add different microneedle extracts to 600uL of 1×10^7^cfu/mL bacterial solution, and divide them into 5 groups, with 2 in each group. Five of them were not treated, and the other 5 of them were subjected to NIR irradiation (1.5Wcm^-2^, 808nm, 5min). Leave and cultivate in an incubator at 37 degrees Celsius for 24 hours to 48 hours, during which time, observe the formation of biofilms between the samples and the blank group. The bacterial solution was then sucked away and the biofilm remained intact, washed carefully and gently 2-3 times with PBS, then completely sucked up the PBS and dried. 70% methanol was added to fix the biomembrane for 30 minutes, then the fixing solution was sucked out and dried. Crystal violet dye was added. After dyeing for 10 minutes, the crystal violet was sucked away. The crystal violet was washed three times with PBS until no color remained. After air drying, photos were taken to record the experimental results. After taking pictures, the biofilm was dissolved with 75% ethanol for about 5 minutes, and then the biofilm destruction rate was calculated by measuring OD_570_ nm with a microplate reader. % destruction rate =(OD_A_-OD_B_)/OD_A_ *100%, (OD_A_: control group, OD_B_: experimental group).

#### 6.3 CLSM observes living/dead bacterial biofilms

Take different microneedle extractives and PBS solutions, place them in confocal small dishes, and add 600uL of 1×10^7^cfu/mL bacterial solution to the samples. One group of samples of different materials is not processed, and the other group is subjected to NIR irradiation (1.5Wcm^-2^, 808nm, 5min), and incubated in an incubator at 37 degrees Celsius for 24-48 hours. During this period, the formation of biofilm between the samples and the blank group is observed.

The bacterial solution was then sucked away and the biofilm was intact. Carefully and gently washed 2-3 times with PBS. Then, the PBS was completely sucked and dried. Then, DMAO/PI dye diluted 1:1000 was added, stained and cultured in a 37℃ biochemical incubator for 30 minutes, and then the confocal cuvette attached to the biofilm was taken out and imaged with a laser confocal microscope to obtain the 3D structure of the biofilm.

### 7 GOx@ZT-MN prevents apoptosis by clearing ROS

#### 7.1 DCFH-DA experiment

In order to study the intracellular ROS scavenging ability of nanoenzymes, we selected hydrogen peroxide as the representative ROS, seeded L929 cells in a 6-well plate (1*10^4^) and incubated for 24 hours, stimulated with H_2_O_2_ (100μmol ml^-1^) for 12 hours, and then added each group of materials and incubated for 12 hours. Next, cells were washed three times with PBS before adding the ROS-specific probe 2,7-dichlorodihydrofluorescein diacetate (DCFH-DA, 10μM). Finally, the early apoptosis status of cells was analyzed by observing the fluorescent mitochondrial membrane potential in L929 with an inverted fluorescent microscope.

#### 7.2 Mitochondrial membrane potential and living/dead cell staining

After L929 cells were treated as above, the cells treated with H_2_O_2_ and each group of materials were stained using the mitochondrial membrane potential detection kit (JC-1) and the calcein-acetyl methyl ester/propidium iodide (Calcein-AM/PI) staining kit respectively. After incubation and washing, they were observed under laser confocal microscope and inverted fluorescent microscope.

### 8 Ability of GOx@ZT-MN to promote proliferation, migration and angiogenesis in vitro

#### 8.1 Cell proliferation activity

The calcein staining kit was used to conduct cell proliferation activity test. First, L929 cells were planted in a 48-well plate containing high-sugar DMEM medium at a density of 1×10^4^. After adhering to the wall, the samples of each group were co-cultured with the cells for 12 hours and 24 hours. Then the calcein solution was added to the above-mentioned well plate with cells. After incubation for 30 minutes, the stained cells were observed under an inverted fluorescent microscope.

#### 8.2 Wound healing assay

L929 cells were seeded in 6-well plates containing high-sugar DMEM medium and incubated at 37℃ until 90% confluence was achieved. Each well was scraped with a 200μl pipette tip and washed with PBS to remove floating cells. Different microneedles were co-cultured with PBS and high-sugar DMEM medium. Cell migration was observed under an inverted microscope at different time points (0, 12 and 24 hours), and the area of scratch healing was measured by ImageJ.

#### 8.3 Transwell Experiment

The detection of L929 cells by each group of samples was determined through a 24-well cross-well chamber. Inoculate 100μl of cell suspension cells with a density of 5×10^5^ml^-1^ in the upper cavity, and add a mixture containing each group of microneedle extract and equal amounts of PBS and high-sugar DMEM medium to the lower cavity. After a 10-hour incubation period, cells were fixed with 4% formaldehyde for 30 minutes and washed three times in PBS. Stained with 0.1% crystal violet for 15 minutes and washed 3 times with PBS. Migration of cells in the upper compartment was observed using an inverted microscope and quantified using ImageJ software.

#### 8.4 Tube formation experiment

For in vitro angiogenesis assays, 200μl of thawed matrigel per well was evenly spread into a pre-cooled 96-well plate and incubated at 37°C for 1 hour. At the same time, HUVECs were digested and resuspended, and co-cultured with each group of materials for 6 hours. To assess tube formation, images were collected with an inverted microscope and quantitatively analyzed using ImageJ software.

### 9 GOx/ZTMN accelerates MRSA-infect wound healing in vivo

#### 9.1 Establishment of type Ⅱ diabetes wound model and evaluation of healing

C57 mice aged 4-6 weeks were induced to develop insulin resistance by receiving a high-sugar and high-fat diet for 6-8 weeks. Streptozotocin solution was injected into the mice to establish a type Ⅱ diabetes model. To indicate that mice had diabetes, an electronic blood glucose meter was used to monitor blood sugar levels in tail vein blood seven days after injection. Mice with blood sugar levels above 16.7 mmol/L were selected for further study.

To build an infectious wound model, mice were first anesthetized, and a circular uniform incision with a diameter of 8mm was made on the back of the mice. Methicillin-resistant Staphylococcus aureus was then diluted, a cotton swab was applied to the incision and sealed. After holding for 24 hours, obvious inflammatory wounds were obtained. Next, mice were selected for group experiments, and the wound exudate of each mouse was extracted with sterile saline for assessment of wound infection. Near-infrared light irradiation (1.5Wcm^-2^, 808nm, 5min) was performed every day. The wound area of the mice was recorded and photographed every 1, 3, 7, and 14 days. Half of the mice were sacrificed on day 7, and the remaining mice were sacrificed on day14. Tissue around the wound was collected and fixed for histological analysis.

#### 9.2 In vivo experiment of glucose content around the wound of diabetic mouse model

Peripheral blood of the wound of diabetic mice treated with GOx@ZT-MN at different time points (0, 2, 6, 12, and 24h) was collected, and blood glucose level was monitored by glucometer (Yuyue Company, China). Untreated mice were used as controls.

#### 9.3 Measurement of wound tissues H_2_S content

Following modeling, Wound tissues were harvested from the GOx@ZT-MN and control groups at 12, 24, and 36 hours post-operation, with the sampling area expanded 1 cm beyond the incision margin. Tissues were uniformly weighed, minced, and homogenized in pre-chilled 0.01 mol/L phosphate-buffered saline (PBS) at a 1:9 tissue-to-PBS ratio. Homogenates were centrifuged at 3000 × g for 4 minutes at 4 °C, and supernatants were collected for hydrogen sulfide (H₂S) concentration analysis using a sensitive sulfur electrode method.

Briefly, a standard sulfide ion solution and Sulfide Antioxidant Buffer (SAOB, containing 2.35 mol/L sodium hydroxide and 0.27 mol/L ethylenediaminetetraacetic acid) were prepared. The sulfide ion stock solution was serially diluted to 5, 10, 20, 40, 60, and 80 μmol/L to generate a calibration curve. H₂S concentrations in tissue supernatants were calculated based on this standard curve.

#### 9.4 Histopathological analysis

Tissue samples around the wound were collected and fixed in 4% paraformaldehyde for 24 hours. H&E staining is used to correctly observe infected wounds. In addition, Masson staining was used to calculate changes in the healing process through collagen fibers. Immunohistochemical staining of TNF-α, IL-6, and IL10, and immunofluorescent staining of DHE, AGEs, CD86, CD206, CD31, and α-SMA were used to examine the expression of inflammation-related proteins, collagen deposition, vascular regeneration, and macrophage polarization, and photographed by an upright light microscope and a laser confocal microscope.

#### 9.5 RNA Sequencing and Bioinformatics Analysis

Three mice in the control group and three mice in the GOx@ZT-MN treatment group were taken on the seventh day, and the skin tissues were frozen in liquid nitrogen and sent to Qingke Biological Company for RNA Sequencing and Bioinformatics Analysis.

#### 9.6 RT-qPCR

Total RNA was extracted from mouse skin tissue from GOx@ZT-MN and control mice using an animal RNA isolation kit and reverse transcribed to obtain cDNA, and then RT-qPCR was performed in a total volume of 20μL according to the manufacturer's instructions. RT-qPCR uses the primer set in Table S1.

### 10 Statistical analysis

In this study, GraphPadPrism10 was used for statistical analysis and charting of all experimental results, which were expressed in the form of mean ± standard deviation (Mean±SD), and differences among multiple groups of continuous variables were used for one-way analysis of variance. All experimental results were repeated more than 3 times (including the CCK-8 experiment was repeated more than 6 times), and the differences in the following p-values were considered to be statistically significant: *p<0.05, ** p <0.01, ***p <0.001, ****p<0.0001.

# **Support figures**


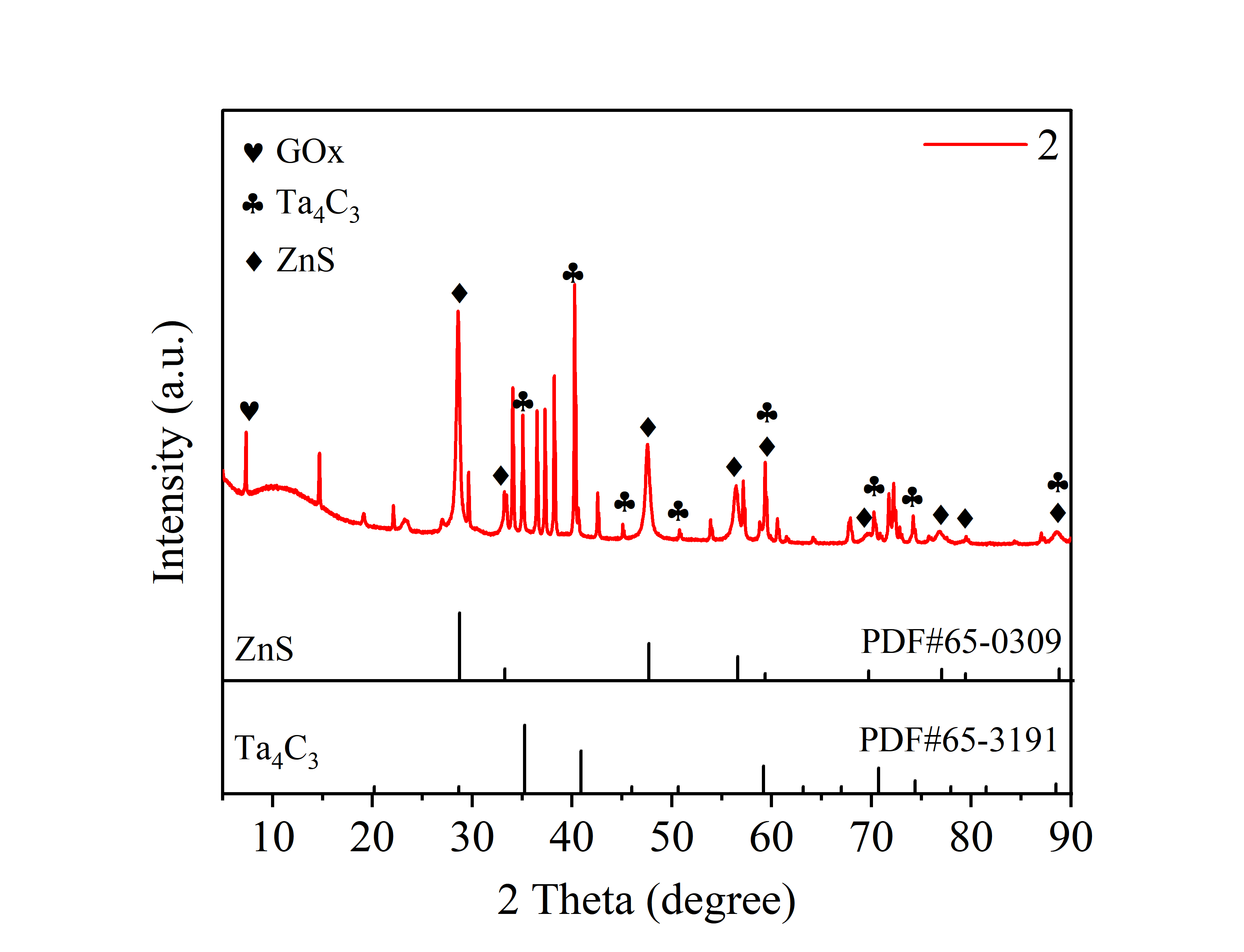


**Fig. S1.** X-ray diffraction analysis of GOx, ZnS and Ta_4_C_3_.


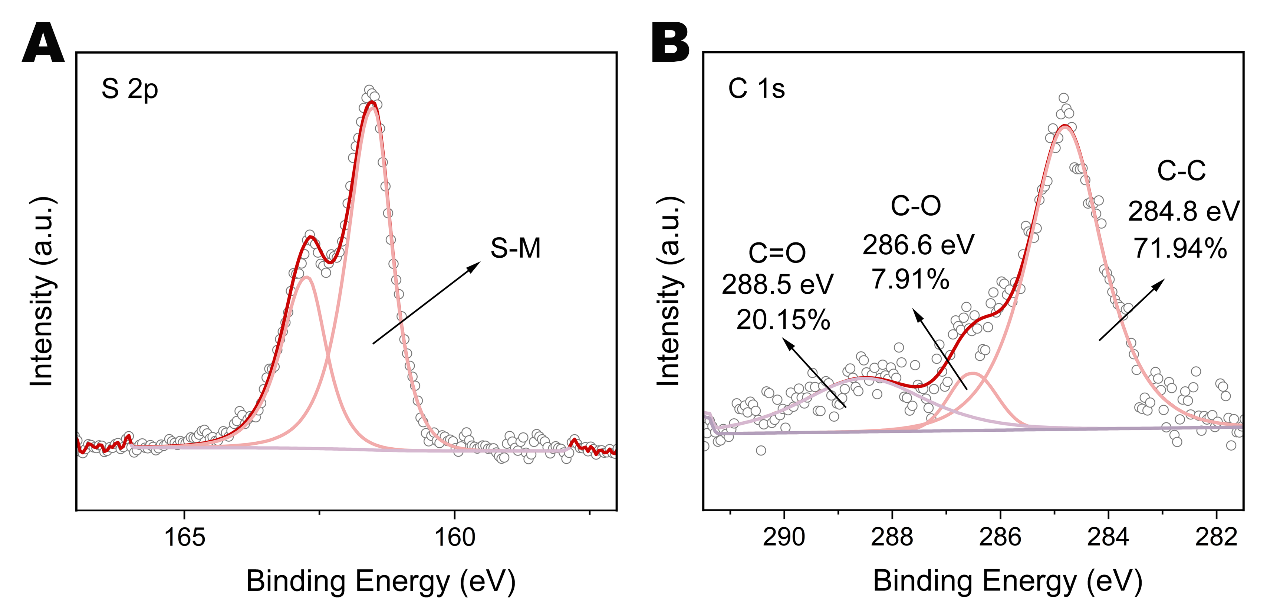


**Fig. S2.** (A) High-resolution S 2p XPS spectra of GOx@ZT. (B) High-resolution C 1s XPS spectra of GOx@ZT.


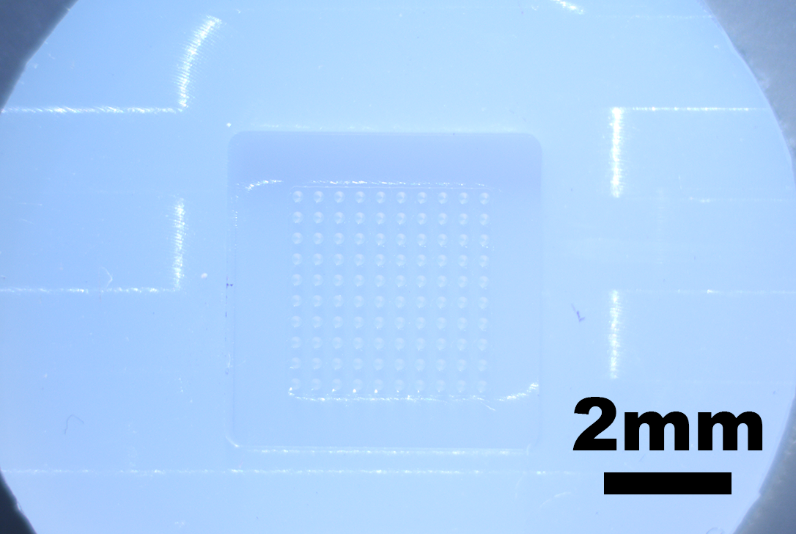


**Fig. S3.** Photo of PDMS microneedle mold. (Scale bar = 2mm)


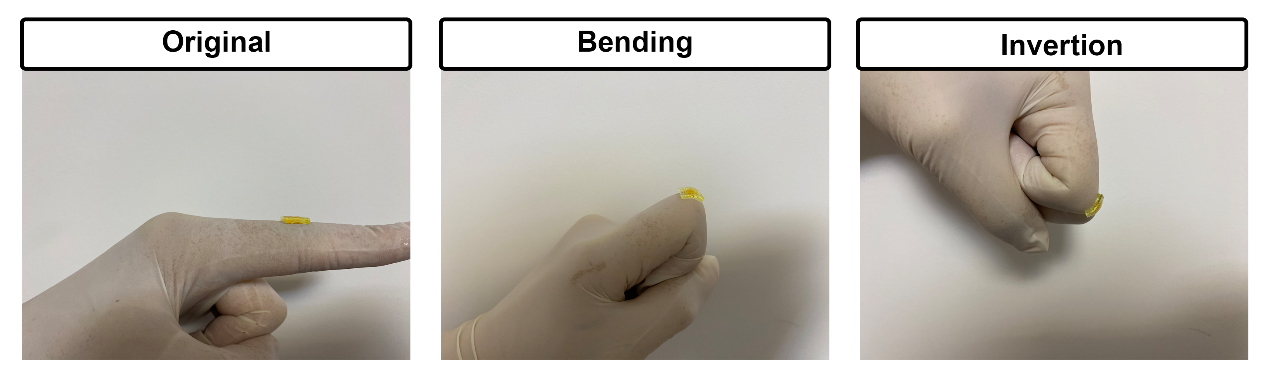


**Fig. S4.** The adhesiveness of GOx@ZT-MN


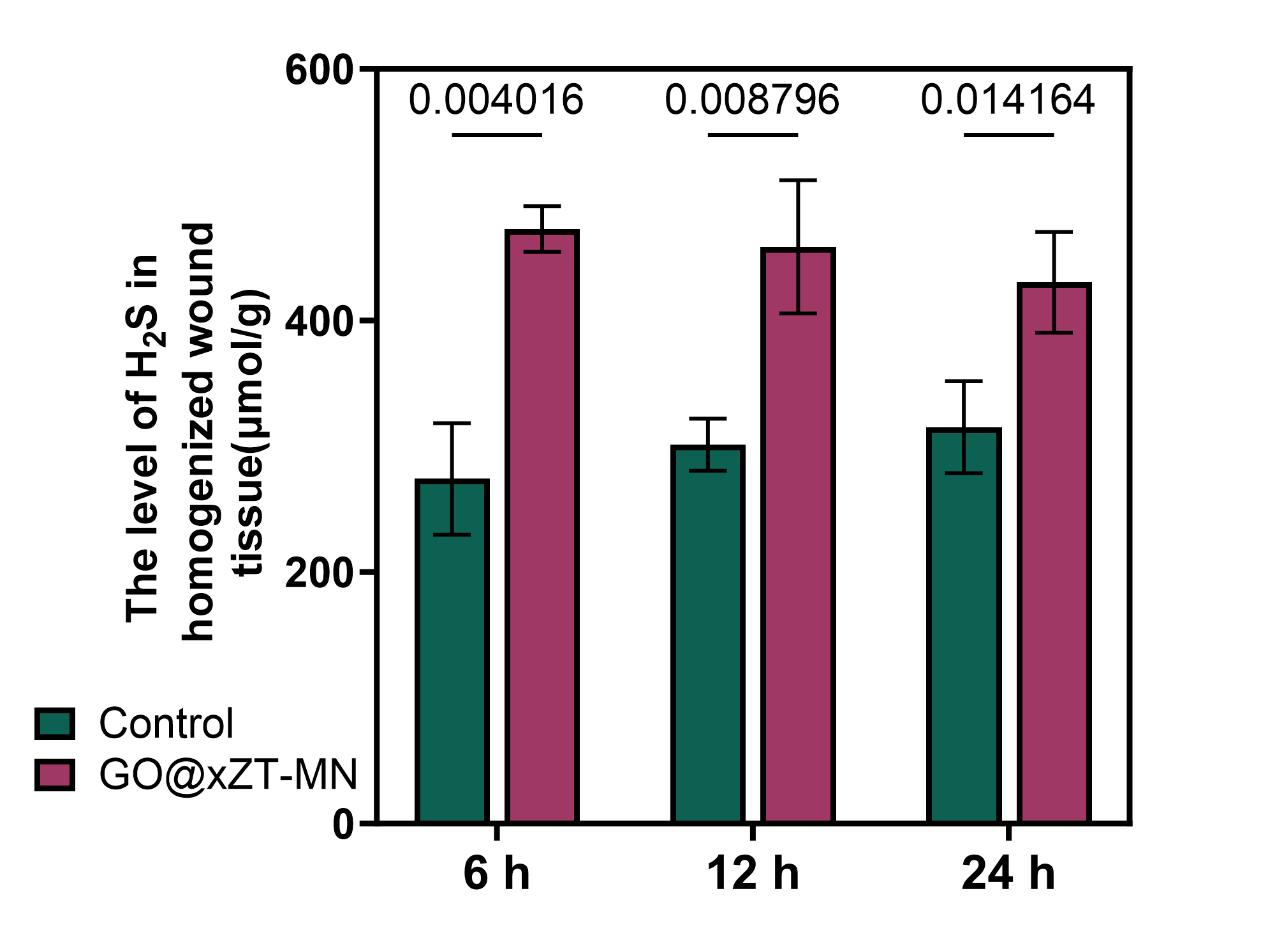


**Fig. S5.** Quantification of H_2_S.in homoge. Data are presented as mean ± SD (n = 3).


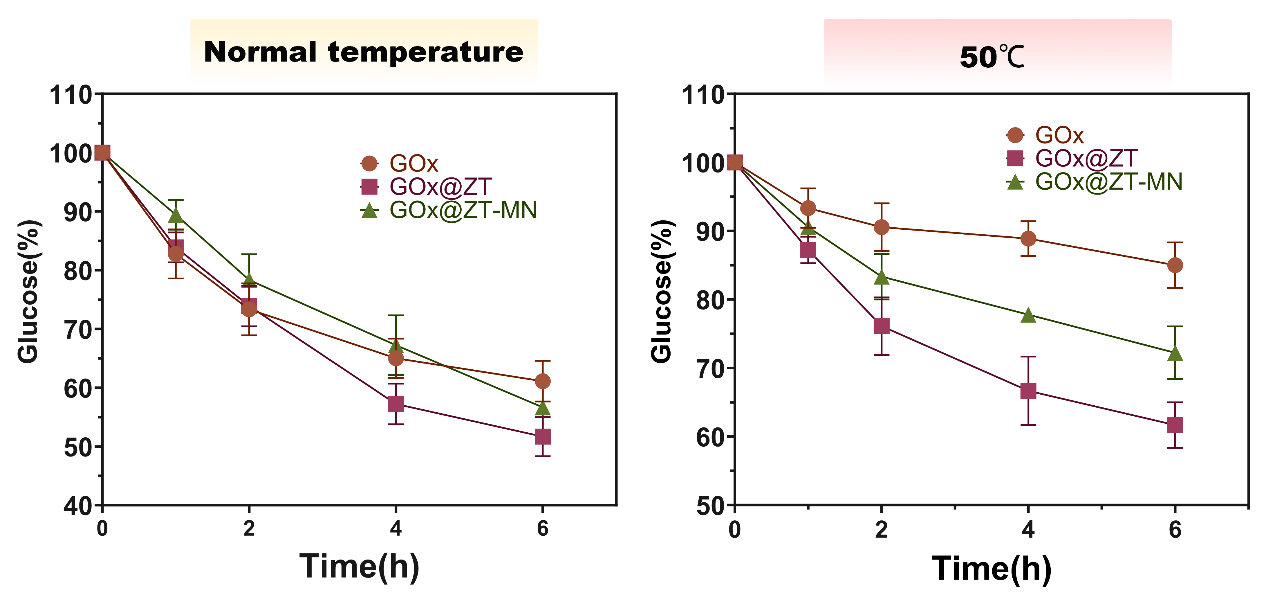
**Fig. S6.** Quantification of GOx enzyme activity in equal amounts of GOx, GOx@ZT and GOx@ZT-MN at different temperatures. Data are presented as mean ± SD (n = 3). ^*^*P* < 0.05, ^**^*P* < 0.01, ^***^*P* < 0.001, ^****^*P* < 0.0001. ns: no significant difference.

**
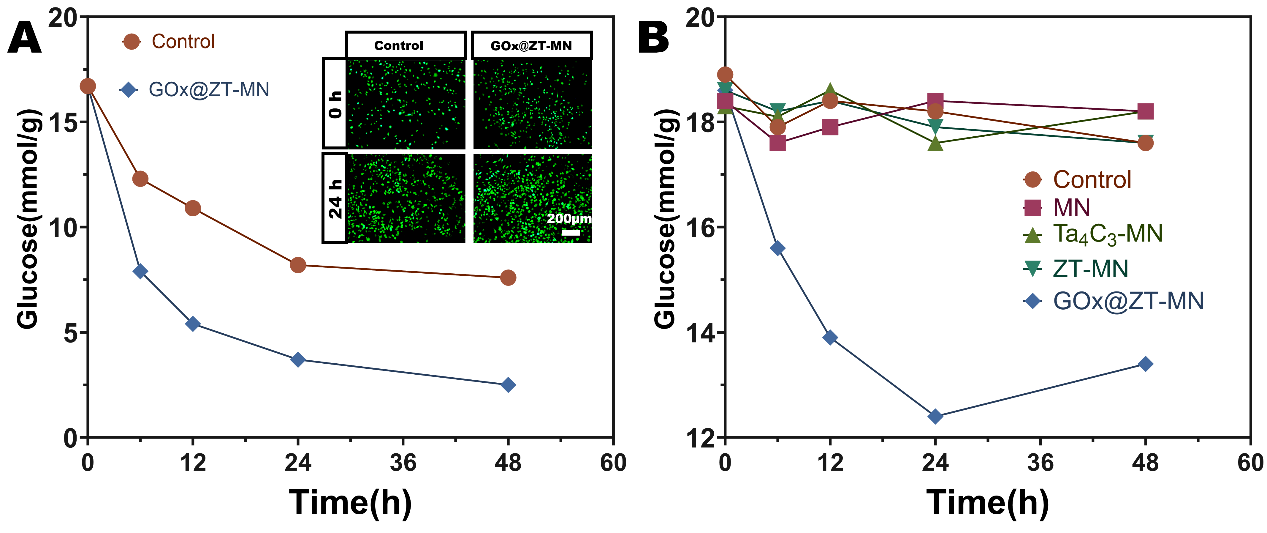
**

**Fig. S7.** Glucose content changes in vitro and Calcein-AM staining of L929 cells after immersion with microneedles. (B) Glucose content changes in the wound skin tissue of diabetic mice after treatment with different treatment groups over 48 h.


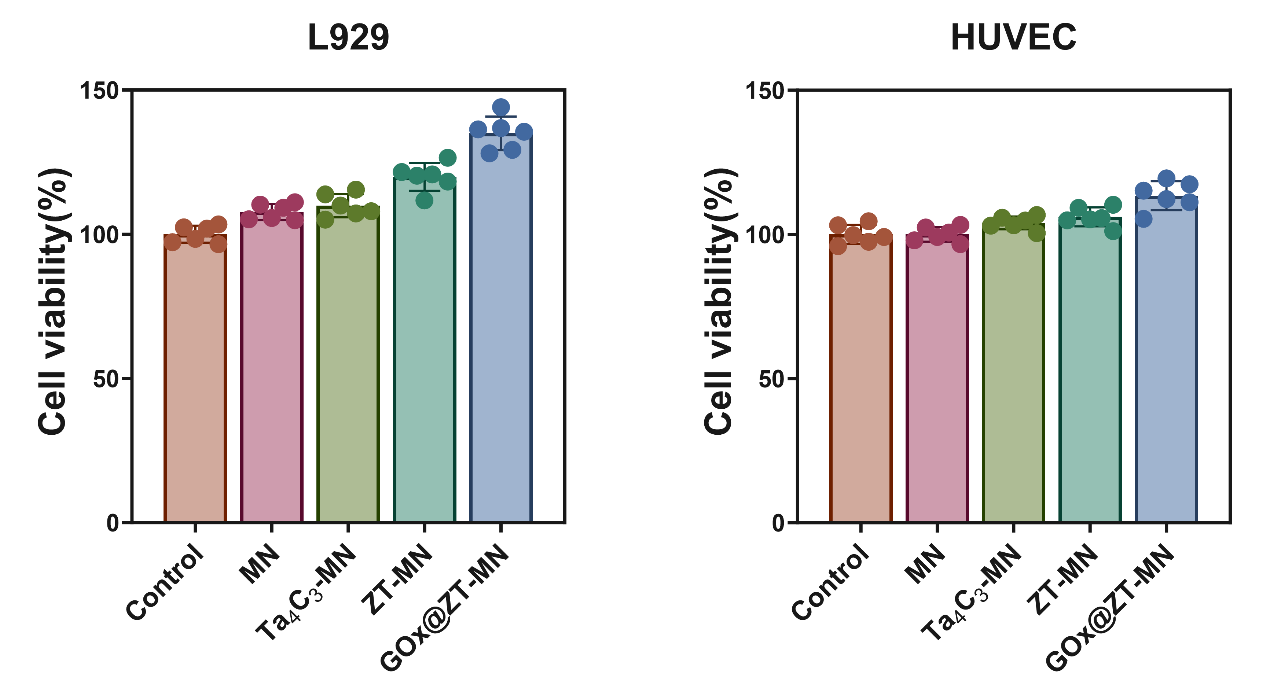
**Fig. S8.** Quantitative analysis of cell viability in L929 cells and HUVECs(n=6). Data are presented as mean ± SD (n = 3). ^*^*P* < 0.05, ^**^*P* < 0.01, ^***^*P* < 0.001, ^****^*P* < 0.0001. ns: no significant difference.


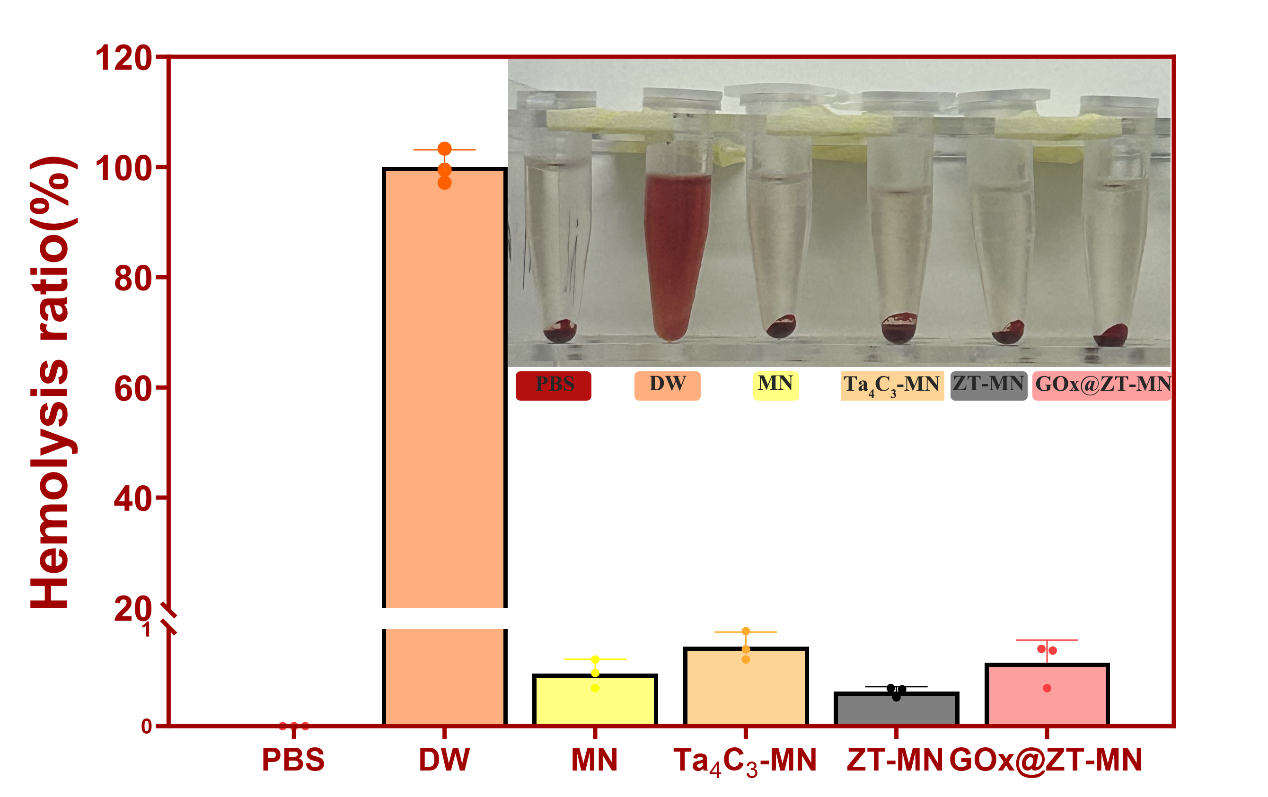
**Fig. S9.** Quantitative analysis and pictures of PBS, DW, Control, MN, Ta_4_C_3_-MN, ZT-MN and GOx@ZT-MN hemolysis experiments


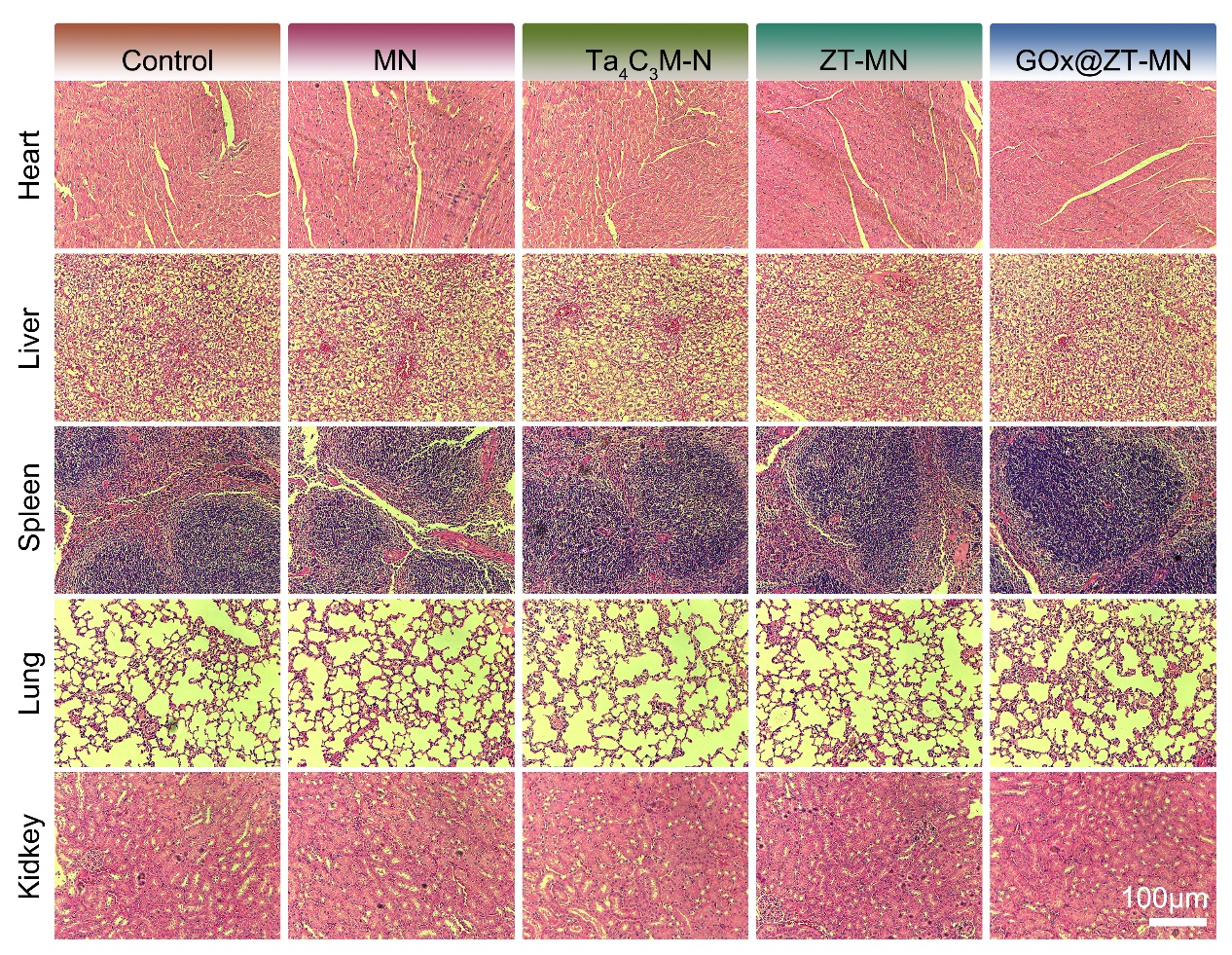
**Fig. S10.** H&E staining of heart, liver, spleen, lung, and kidney of mice treated with Control, MN, Ta_4_C_3_-MN, ZT-MN and GOx@ZT-MN groups for half a month. (Scale bar = 100μm)


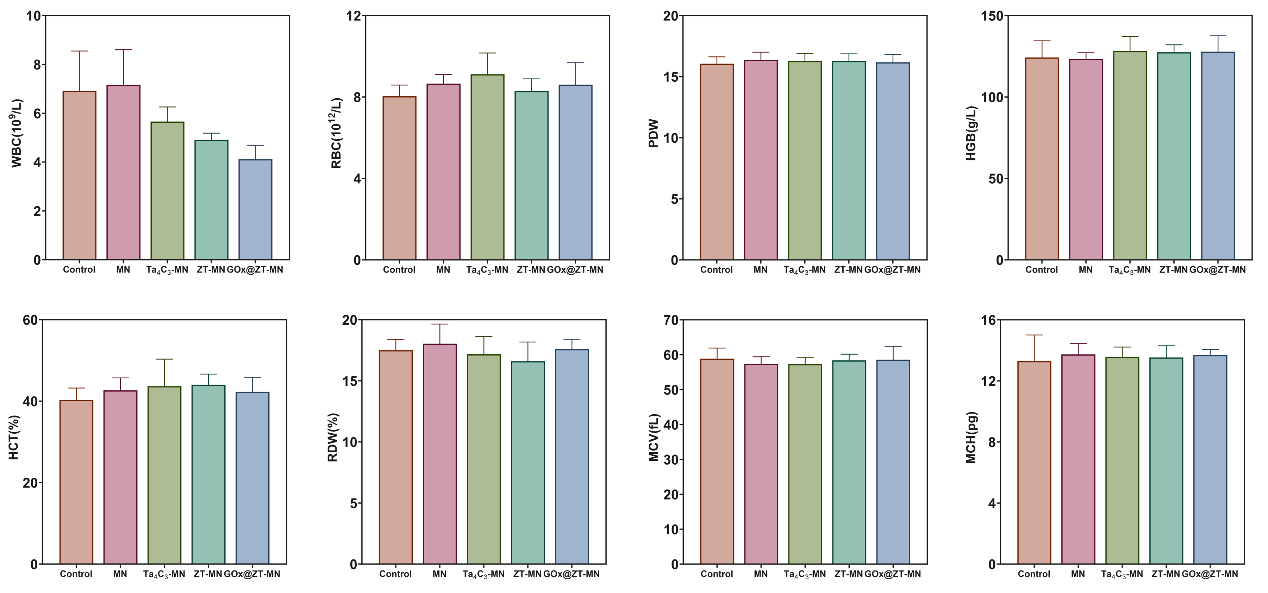


**Figure S11.** Evaluation of blood routine indexes in diabetic mice (n=3).


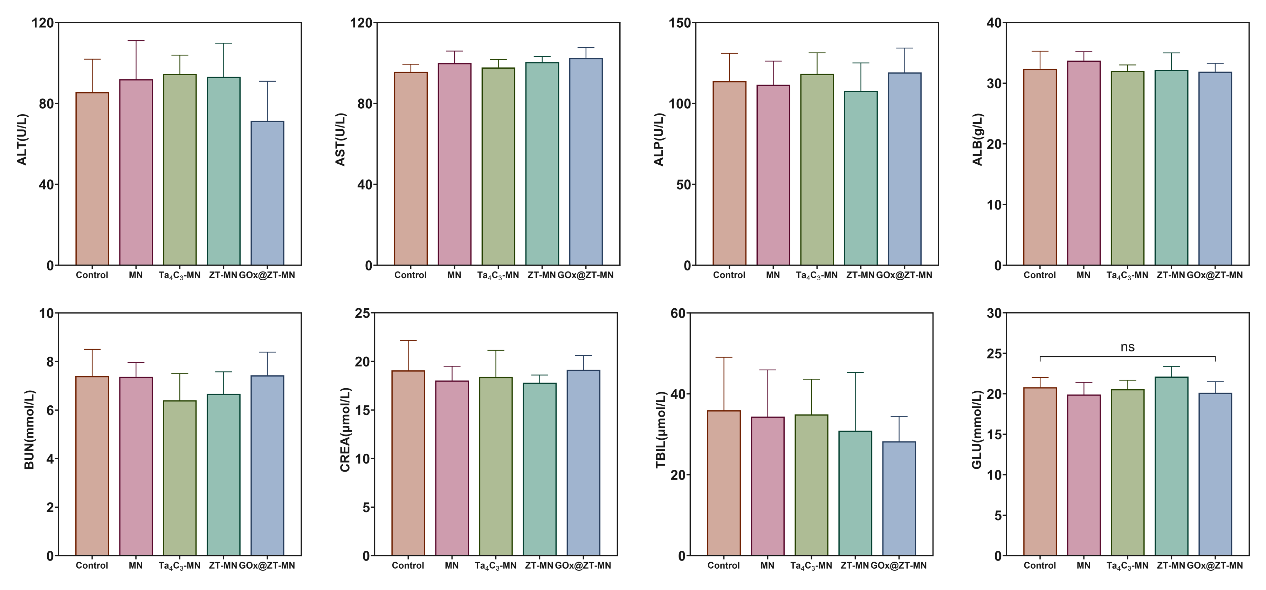


**Figure S12.** Evaluation of blood biochemical indexes in diabetic mice (n=3).


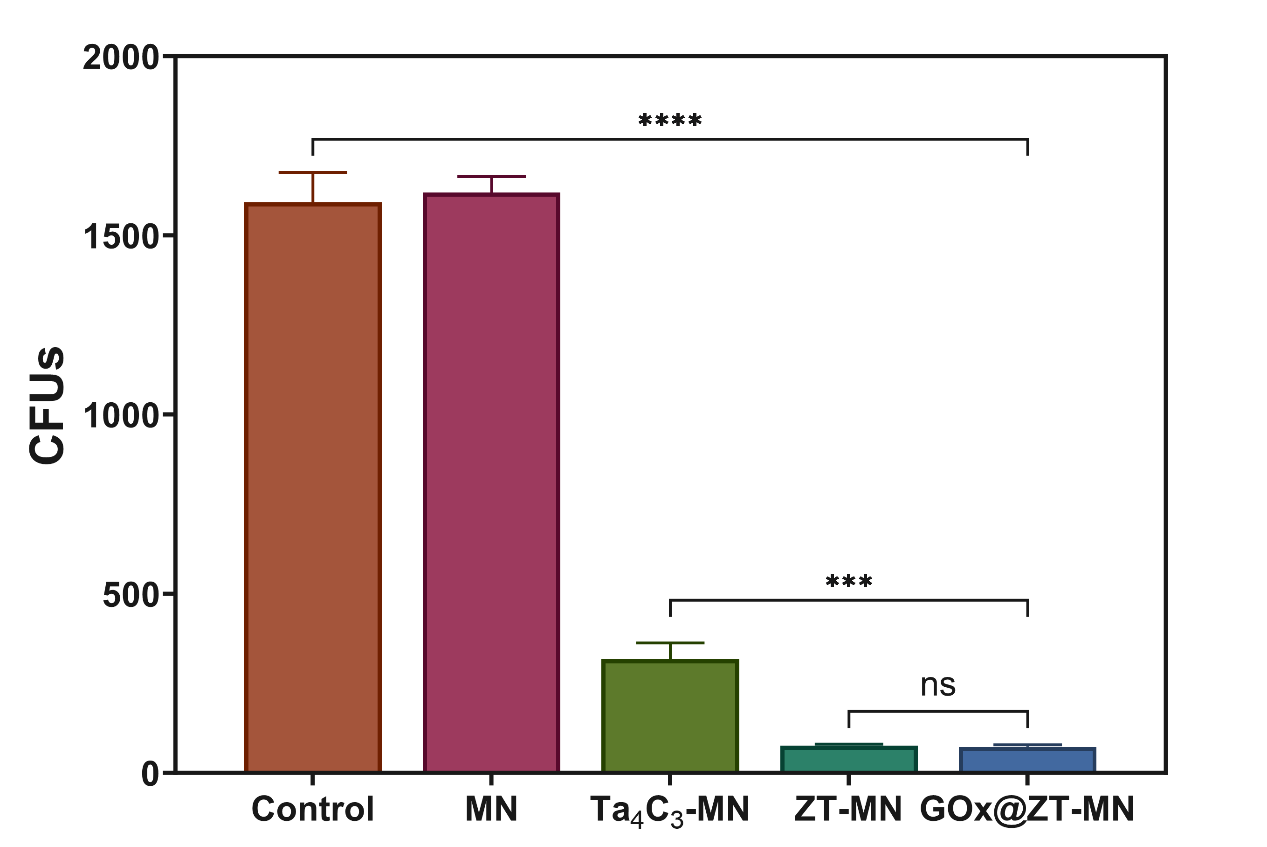


**Fig. S13.** Quantitative Analysis of MRSA in Wound Samples Following Control, MN, Ta_4_C_3_-MN, ZT-MN and GOx@ZT-MN groups Treatment in an Infected Diabetic Mouse Model. Data are presented as mean ± SD (n = 3). ^*^*P* < 0.05, ^**^*P* < 0.01, ^***^*P* < 0.001, ^****^*P* < 0.0001. ns: no significant difference.


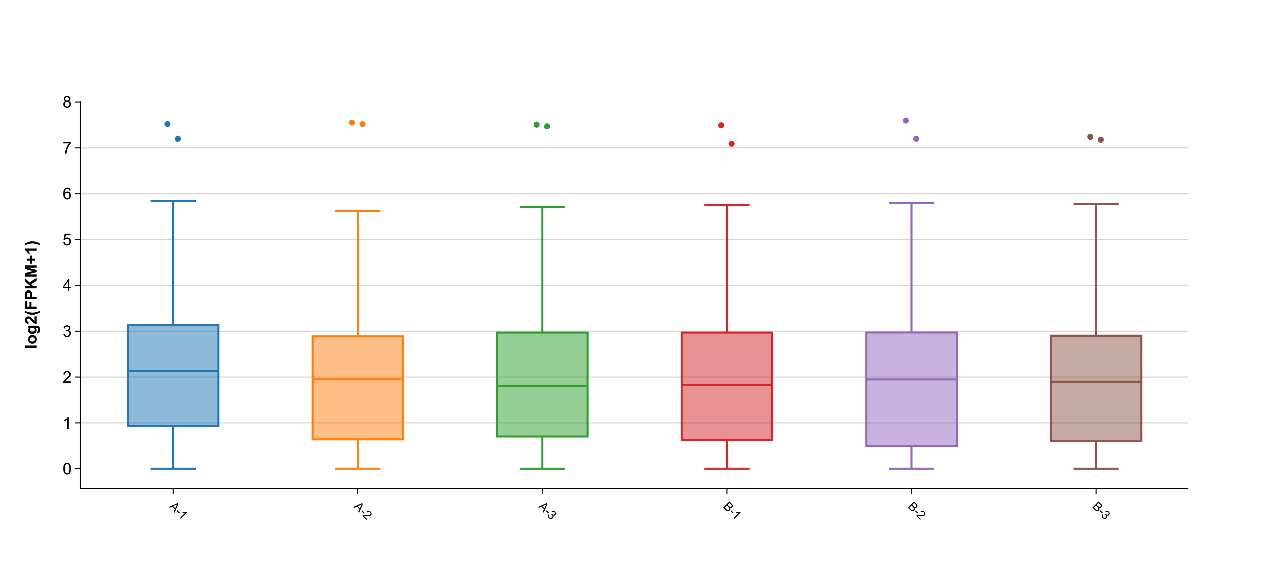


**Fig. S14.** Box type drawing of gene expression levels in each sample. (A; Control group, B: GOx@ZT-MN group)

**
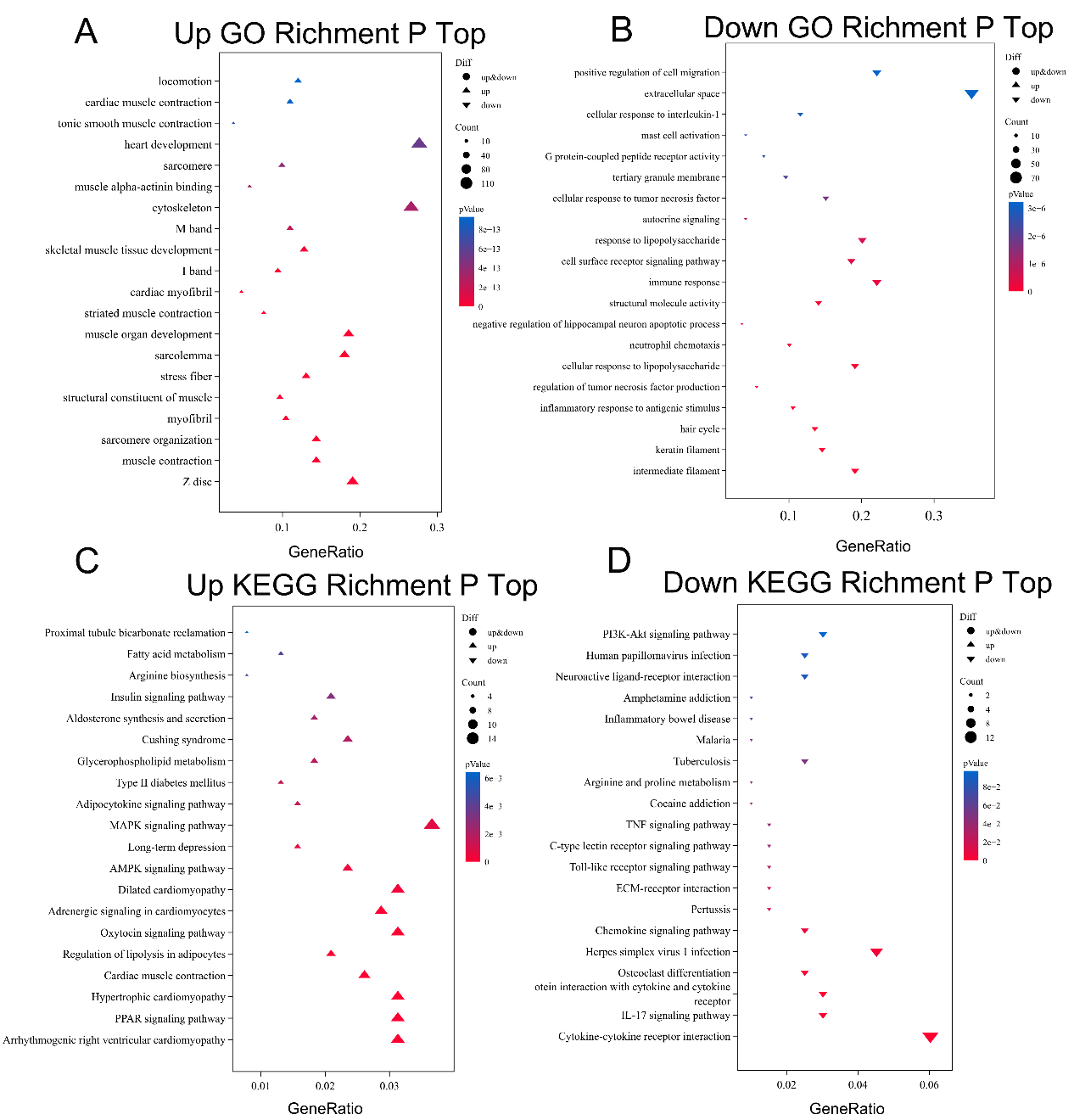
**

**Fig. S15.** GO and KEGG enrichment diagrams (A) Up-regulated the GO enrichment diagrams. (B) Down-regulated the GO enrichment diagram. (C) Upregulated KEGG enrichment pattern. (D) Down-regulated KEGG enrichment pattern.

**Table. S1.** Primers sequences for RT-qPCR

| **Gene** | **Primer sequence (5’ to 3')** | **Product Size (bp)** |
| --- | --- | --- |
| 1-VEGFa-F3 | ATTTATTGGTGCTACTGTTTATCCG | 95 |
| 1-VEGFa-R3 | CGGAATATCTCGGAAAACTGCT |  |
| 2-HIF-1α-F3 | TTGGTTTGATTCTGGTACATGGC | 157 |
| 2-HIF-1α-R3 | GCTGGAAGGTTTGTGGTGTTT |  |
| 3-IL6-F2 | CACAGAAGGAGTGGCTAAGGA | 101 |
| 3-IL6-R2 | GCACTAGGTTTGCCGAGTAGA |  |
| 4-TNFα-F3 | GGCCTTCCTACCTTCAGACC | 197 |
| 4-TNFα-R3 | CTGAAGACAGCTTCCCACACT |  |
| 5-FGF1-F2 | GATATGGAGCTGGGTGTATCCC | 110 |
| 5-FGF1-R2 | TGCCTTCAACACAAAGCAGG |  |
| GAPDH-F | ACTCTTCCACCTTCGATGCC | 193 |
| GAPDH-R | TGGGATAGGGCCTCTCTTGC |  |
